# An EMT–primary cilium–GLIS2 signaling axis regulates mammogenesis and claudin-low breast tumorigenesis

**DOI:** 10.1126/sciadv.abf6063

**Published:** 2021-10-27

**Authors:** Molly M. Wilson, Céline Callens, Matthieu Le Gallo, Svetlana Mironov, Qiong Ding, Amandine Salamagnon, Tony E. Chavarria, Roselyne Viel, Abena D. Peasah, Arjun Bhutkar, Sophie Martin, Florence Godey, Patrick Tas, Hong Soon Kang, Philippe P. Juin, Anton M. Jetten, Jane E. Visvader, Robert A. Weinberg, Massimo Attanasio, Claude Prigent, Jacqueline A. Lees, Vincent J. Guen

**Affiliations:** 1 Koch Institute for Integrative Cancer Research at MIT, Cambridge, MA, USA.; 2 Department of Biology, Massachusetts Institute of Technology, Cambridge, MA, USA.; 3 Institut de Génétique et Développement de Rennes, Centre National de la Recherche Scientifique, Rennes, France.; 4 INSERM U1242, Rennes 1 University, Rennes, France.; 5 Centre de Lutte Contre le Cancer Eugène Marquis, Rennes, France.; 6 Department of Internal Medicine, University of Iowa Carver College of Medicine, Iowa City, IA, USA.; 7 Plateforme d’Histopathologie de Haute Précision (H2P2), Rennes, France.; 8 Department of Biological Engineering, Massachusetts Institute of Technology, Cambridge, MA, USA.; 9 Cell Biology Section, Division of Intramural Research, National Institute of Environmental Health Sciences, National Institutes of Health, Research Triangle Park, NC, USA.; 10 CRCINA, INSERM, Université de Nantes, Nantes, France.; 11 Stem Cells and Cancer Division, The Walter and Eliza Hall Institute of Medical Research and Department of Medical Biology, The University of Melbourne, Parkville, Victoria, Australia.; 12 MIT Department of Biology and the Whitehead Institute, Cambridge, MA, USA.; 13 CRBM, CNRS, Université de Montpellier, Montpellier, France.

## Abstract

The epithelial-mesenchymal transition (EMT) and primary ciliogenesis induce stem cell properties in basal mammary stem cells (MaSCs) to promote mammogenesis, but the underlying mechanisms remain incompletely understood. Here, we show that EMT transcription factors promote ciliogenesis upon entry into intermediate EMT states by activating ciliogenesis inducers, including FGFR1. The resulting primary cilia promote ubiquitination and inactivation of a transcriptional repressor, GLIS2, which localizes to the ciliary base. We show that GLIS2 inactivation promotes MaSC stemness, and GLIS2 is required for normal mammary gland development. Moreover, GLIS2 inactivation is required to induce the proliferative and tumorigenic capacities of the mammary tumor–initiating cells (MaTICs) of claudin-low breast cancers. Claudin-low breast tumors can be segregated from other breast tumor subtypes based on a GLIS2-dependent gene expression signature. Collectively, our findings establish molecular mechanisms by which EMT programs induce ciliogenesis to control MaSC and MaTIC stemness, mammary gland development, and claudin-low breast cancer formation.

## Teaser

EMT induces cilium assembly and signaling to promote mammogenesis and tumorigenesis.

## INTRODUCTION

The mammary gland is a branched ductal tissue composed of a stratified epithelium containing luminal and basal cells surrounded by a basement membrane embedded in stroma (*1*). Mammary gland development is fueled by basal multipotent and lineage-restricted unipotent mammary stem cells (MaSCs) (*1*). The stem cell properties of basal MaSCs are induced by epithelial-mesenchymal transition (EMT), a cell-biological program that enables epithelial cells to acquire an array of mesenchymal phenotypes (*2*–*5*). The EMT transcription factor (EMT-TF) Slug is expressed in MaSC-enriched basal cells (*2*–*4*, *6*, *7*). The ability of these stem cells to form organoids and reconstitute the mammary epithelium in transplantation experiments is enhanced or impaired by Slug overexpression or loss, respectively (*3*, *4*, *7*). Consistent with these findings, Slug-knockout (KO) mice demonstrate delayed mammary gland development (*6*).

Breast cancer comprises a variety of tumor types that are classified into therapeutic and molecular subtypes (*8*). The triple-negative breast cancer (TNBC) therapeutic subtype, in particular, is composed of breast tumors that do not express nuclear hormone receptors and HER2 and includes the less differentiated claudin-low molecular subtype (*8*). Claudin-low breast tumors are characterized by low expression of epithelial cell-cell adhesion genes and high expression of EMT and basal MaSC genes (*8*). In murine carcinoma models of claudin-low breast cancer, the tumorigenic activity of mammary tumor–initiating cells (MaTICs) relies on EMT-TFs (*2*, *3*, *9*–*11*). Collectively, these studies establish that EMT programs tightly regulate mammary gland development and tumorigenesis by controlling both MaSCs and MaTICs.

Primary ciliogenesis is a dynamic process in which a single microtubule-based structure, the primary cilium, is assembled by a modified version of the mother centriole, called the basal body, at the plasma membrane and protrudes from the surface, where it acts as a cell signaling center (*12*). Primary ciliogenesis requires the coordination of many cellular processes including a motor-driven process that involves the bidirectional movement of multiprotein complexes along the cilium, called intraflagellar transport (IFT) (*12*). Primary cilia control a variety of signaling pathways, including the Hedgehog pathway, to regulate cell fate specification and tissue development (*13*). Components of signaling pathways are enriched and processed at the posttranscriptional level within the primary cilium (*13*). In the context of numerous tissues, including the brain (*14*, *15*), mammary gland (*5*, *11*), adipose tissue (*16*), and olfactory epithelium (*17*), primary cilia play a key role in modulating stem cell self-renewal versus differentiation. We recently showed that EMT programs promote primary ciliogenesis in MaSC and MaTIC and that these primary cilia are required for stemness in these cell populations (*11*). However, the molecular mechanisms linking EMT, primary cilia, and stemness in the mammary gland are largely unknown.

Here, we show that EMT programs induce primary ciliogenesis upon entry into intermediate EMT states in both human and mouse mammary glands. We establish that EMT-TFs induce the expression of direct ciliogenesis regulators and IFT inducers, including fibroblast growth factor receptor 1 (FGFR1), to promote primary ciliogenesis. We show that primary cilia control ubiquitination and inactivation of a key central signaling node and transcriptional repressor, GLIS2 (GLIS family zinc finger 2), which localizes to the ciliary base. Furthermore, we demonstrate that GLIS2 inactivation promotes the stemness of MaSCs and MaTICs, mammogenesis, and claudin-low breast tumorigenesis.

## RESULTS

### EMT programs induce primary ciliogenesis upon entry into intermediate transition states in the mammary gland

**Fig. 1.**
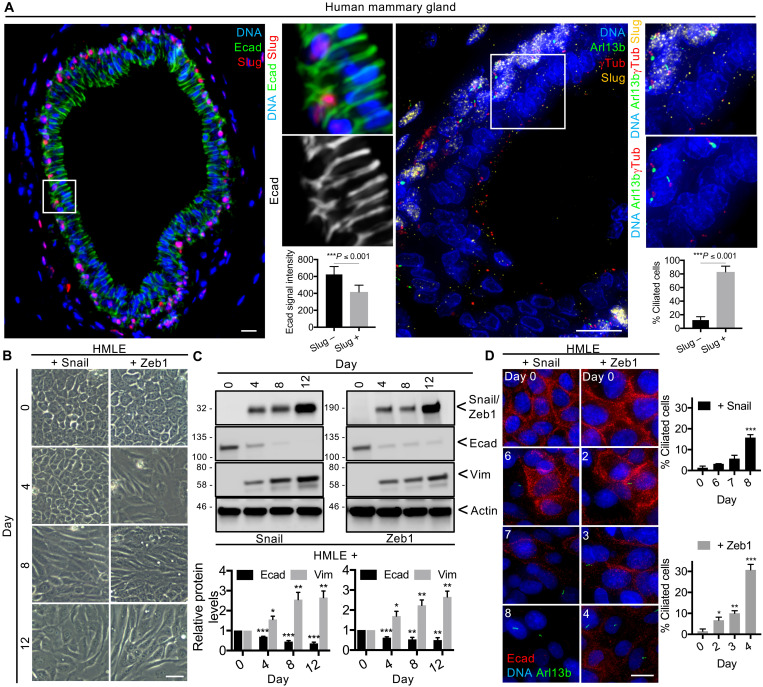
EMT programs induce primary ciliogenesis upon entry into intermediate transition states. (**A**) Mammary gland sections from healthy human patients were stained for the indicated proteins (inset: 3×, *n* = 4). E-cadherin expression (Ecad signal intensity on Slug^+^ basal-basal cell junctions or Slug^−^ luminal-luminal cell junctions) and the percentage of ciliated cells were quantified. Representative results from three independent experiments are shown. (**B** to **D**) Epithelial-like HMLE cells were treated with doxycycline (1 μg/ml) to induce Snail or Zeb1 expression over 12 days. (B) The morphology of the cells was examined by brightfield microscopy, (C) their expression of the indicated proteins was analyzed by Western blot, protein levels were quantified (*n* = 3, means ± SEM), (D) their ability to form primary cilia was determined, and the percentage of ciliated cells was quantified at the indicated time points (*n* = 3, means ± SEM). Representative results from three independent experiments are shown. All scale bars, 50 μm (brightfield) and 15 μm (immunofluorescence). *P ≤ 0.05; **P ≤ 0.01; ***P ≤ 0.001.

We previously established that EMT programs are associated with primary ciliogenesis in the mouse mammary gland (*11*), spurred by our discovery that primary cilia were present in a high fraction of basal Slug-expressing cells present within ducts and terminal end buds in the murine mammary epithelium of a Slug-IRES-YFP mouse (fig. S1A) (*11*). To determine whether this was also true in the human gland, we analyzed sections of human reduction mammoplasty tissue. We detected Slug^+^ cells specifically in the basal Krt14^+^ layer of the human mammary epithelium and found that these cells expressed significantly lower levels of the epithelial marker E-cadherin than did the Slug^−^ luminal cells (Fig. 1A and fig. S1, B and C). Primary cilia (detected by the cilium marker Arl13b and the centrosome marker γTubulin) marked the vast majority of Slug-expressing cells, in contrast to the Slug-negative cells (Fig. 1A). Hence, basal mammary epithelial cells, which exist in an intermediate EMT state, assemble primary cilia in both human and mouse mammary glands.

To determine whether primary ciliogenesis is a transient or a stable response to EMT activation, we used HMLE cells, which are immortalized human mammary epithelial cells (harvested from a mammoplasty and expressing hTERT and SV40 large T proteins) (*18*). We previously generated HMLE variants in which doxycycline induces ectopic expression of the Snail or Zeb1 EMT-TFs (*11*). To eliminate preexisting mesenchymal cells from the uninduced HMLE variant populations, we used fluorescence-activated cell sorting (FACS) to isolate the most epithelial cells based on their CD44^Lo^;CD24^Hi^ phenotype (fig. S2A) (*2*). We then tested our ability to induce EMT and detect various EMT states in these epithelial-enriched populations by culturing them with doxycycline for 12 days and examining their morphology and expression of epithelial (E-cadherin) and mesenchymal (vimentin) markers at various time points. At day 0, both HMLE variants displayed the classic epithelial-like (E-like) cobblestone morphology (Fig. 1B) and lacked detectable expression of either Snail or Zeb1 (Fig. 1C). Doxycycline treatment induced Snail and Zeb1 expression with similar kinetics, both appearing by day 4 at similar levels (Fig. 1C and fig. S2B). Despite these similarities, the two variant populations showed differences in their EMT kinetics; the Snail-expressing cells progressively adopted an elongated mesenchymal-like (M-like) morphology between days 8 and 12, while the Zeb1-expressing cells gained an M-like appearance as early as day 4 and became more distinctly mesenchymal at later time points (Fig. 1B). Acquisition of these M-like phenotypes was concurrent with E-cadherin loss, which was only partial at day 4, and more pronounced at later time points in Snail-expressing cells, but was already largely lost 4 days after Zeb1 induction (Fig. 1C). The mesenchymal marker vimentin increased gradually over 12 days in both HMLE variants (Fig. 1C).

Having defined the time windows in which the different EMT states occurred, we next assessed the representation of primary cilia across these states by staining cells for Arl13b alongside E-cadherin. We observed a significant increase in primary ciliogenesis in response to Snail (day 8) or Zeb1 (starting at day 2 and increasing markedly on day 4) expression (Fig. 1D and fig. S2C), which coincided with the marked reduction of E-cadherin, intermediate expression levels of vimentin, and a partial M-like phenotype (Fig. 1, B to D). Together, these data show that EMT programs induce primary ciliogenesis upon entry into intermediate EMT states.

### EMT-TFs activate the expression of positive regulators of cilium assembly to promote primary ciliogenesis

**Fig. 2.**
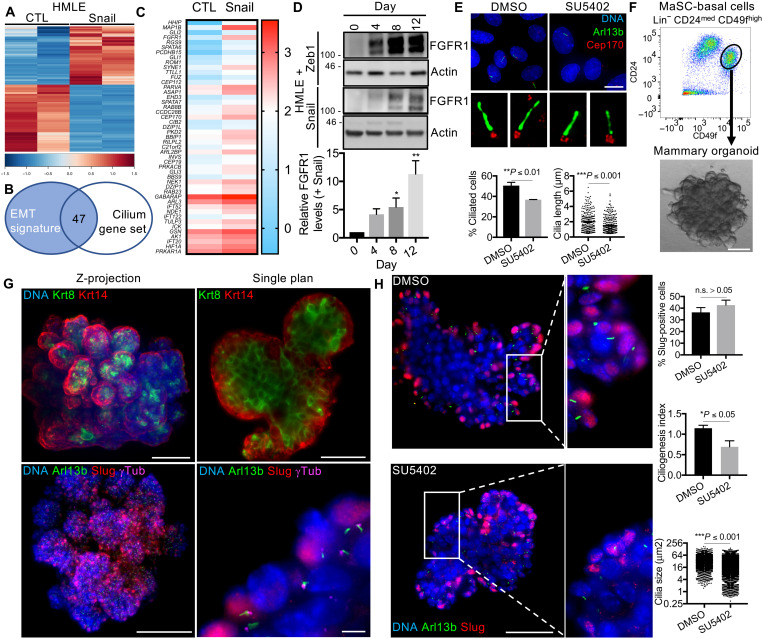
EMT-TFs activate the expression of positive regulators of cilium assembly to promote primary ciliogenesis. (**A**) Gene expression was analyzed in the indicated HMLE variants by RNA-seq. Heatmap showing the differential expression of genes (*q* ≤0.05, fold change ≥2). (**B**) Venn diagram displaying the overlap between the EMT signature [defined in (A)] and a curated cilium gene set from MSigDB. Forty-seven genes were found in both gene sets. (**C**) Heatmap showing the differential expression of the 47 genes in HMLE variants. (**D**) FGFR1 protein expression in the indicated HMLE variants was analyzed by Western blot and quantified (*n* = 3, means ± SEM). Representative results from three independent experiments. (**E**) HMLE + Snail cells were treated with DMSO or SU5402 (10 μM) and stained for the indicated proteins to quantify ciliogenesis (*n* = 3, means ± SEM). Representative results from three independent experiments (inset: 10×; scale bar, 15 μm). (**F** to **H**) Mouse MaSC-enriched basal cells were isolated by FACS and plated in 3D to generate organoids, which were stained for the indicated proteins. Scale bars, 100 μm (top and bottom left panels), 50 μm (top right panel), and 5 μm (bottom right panel). (H) Organoids treated with DMSO or SU5402 (10 μM) were stained for the indicated proteins. Percentage of Slug^+^ cells, ciliogenesis index (Arl13b/Slug particles), and cilia size were determined (*n* = 3, means ± SEM). Representative results from three independent experiments. Scale bar, 50 μm (inset: 2.5×).

The mechanisms by which EMT programs induce primary ciliogenesis have been elusive. To determine the underlying molecular mechanisms, we conducted comparative analyses of control (CTL) and Snail-expressing HMLE cells under ciliogenesis-permissive conditions. To do so, CTL and Snail cells were grown to high confluence and serum-starved. Replicate samples were subjected to immunofluorescent staining (fig. S3A) or Western blotting (fig. S3B), which confirmed that the Snail-expressing cells were highly ciliated relative to CTL cells (fig. S3A), and expressed lower levels of E-cadherin and higher levels of fibronectin, N-cadherin, vimentin, and the EMT-TFs Zeb1 and Twist1 (fig. S3B). Parallel replicate samples were subjected to RNA-sequencing (RNA-seq). This revealed substantial differences in gene expression in CTL versus Snail-expressing cells (Fig. 2A). To identify ciliogenesis regulators whose expression was induced upon EMT induction by Snail, we compared the list of up-regulated genes in Snail-expressing versus CTL cells to a cilium gene set, consisting of genes encoding centrosomal and/or ciliary proteins (combined and curated MSigDB GO_Cilium, GO_Cilium Morphogenesis gene sets). We identified 47 genes that overlapped between the two gene signatures, which then represented candidate ciliogenesis inducers and/or ciliary signaling regulators downstream of Snail (Fig. 2, B and C).

We reasoned that this gene set likely included both direct and indirect Snail targets. To identify potential direct Snail target genes, we reanalyzed the data from an existing Snail chromatin immunoprecipitation sequencing (ChIP-seq) study in mouse mammary tumor cells (*4*) and compared the resulting gene list to our candidate ciliogenesis inducers and cilia components. Of the 47 genes in our list, 34 were Snail targets from the ChIP-seq (table S1). One of the top hits was *Fgfr1*. Multiple binding motifs associated with Snail-dependent transcriptional regulation were identified in close proximity to or within the *FGFR1* promoter (fig. S3C), consistent with the notion that *FGFR1* is directly activated by Snail.

Over the last decade, FGFR1 has emerged as a key inducer of ciliogenesis in the embryonic tissues of lower organisms, including the zebrafish, xenopus, and chick (*19*, *20*), as well as in human lung carcinoma and rhabdomyosarcoma cells (*21*). FGFR1 directly controls IFT by inducing expression of IFT system components and enabling their import into the cilium (*19*, *20*). FGFR1 has not been previously linked to ciliogenesis in the mammary gland, but it is known to cooperate with FGFR2 to promote mammary gland development by regulating stemness of MaSCs (*22*).

Given these observations, we hypothesized that EMT programs activate FGFR1 expression to induce ciliogenesis. After validating the up-regulation of *FGFR1* in Snail-expressing versus CTL HMLE cells by real-time quantitative polymerase chain reaction (RT-qPCR) analysis (fig. S3D), we examined FGFR1 protein expression in Snail- and Zeb1-expressing cells at various EMT states using our doxycycline induction scheme. We found that FGFR1 expression was markedly induced by Snail and Zeb1 expression at days 8 and 4, respectively (Fig. 2D), coinciding with the induction of primary ciliogenesis described earlier (Fig. 1D). To determine whether FGFR1 was activated, we screened for the presence of phosphorylated Tyr^653/654^, which is a hallmark of activation. This was detected in Snail-expressing cells (fig. S3E), establishing that FGFR1 is both induced at the transcriptional level and activated.

To directly test the role of FGFR1 in mammary ciliogenesis, we cultured our Snail-expressing HMLE cells in ciliogenesis-permissive conditions in the presence of various concentrations of the small-molecule FGFR tyrosine kinase inhibitor SU5402 or dimethyl sulfoxide (DMSO) vehicle control. Initially, we examined the impact of drug treatment on FGFR1 activity by assessing the phosphorylation status of two known FGFR1 downstream targets, AKT and MEK (mitogen-activated protein kinase kinase). We saw a significant decrease in the phosphorylation of both targets when cells were treated at 10 μM SU5402 (fig. S3F). This drug treatment did not alter cell cycle withdrawal (as judged by FACS quantification of G_2_-M cells; fig. S3G) or the E-M status of the cells (as judged by Western blotting for E-cadherin and vimentin; fig. S3H), which could affect ciliogenesis indirectly. We then asked whether it alters ciliogenesis by staining DMSO- and SU5402-treated cells for Arl13b and Cep170 (a centrosome marker). This revealed that FGFR1 inhibition is associated with a significant decrease in the percentage of cells displaying primary cilia and in the length of the cilia that succeeded in forming (Fig. 2E). To validate the role of FGFR1 in ciliogenesis, we also tested the effect of a dominant-negative form of FGFR1 (FGFR1dn). When expressed in HMLE Snail-expressing cells, FGFR1dn repressed the phosphorylation of MEK (fig. S3I), as expected for inhibition of FGFR1, and also significantly reduced the fraction of cells bearing primary cilia (fig. S3J).

To evaluate the role of FGFR1 in ciliogenesis in a more physiological setting, we next examined the impact of SU5402 treatment on ciliogenesis in mammary organoids. For this, we isolated MaSC-enriched basal cells from adult female mice by FACS based on their Lin^−^;CD24^med^;CD49f^Hi^ phenotype (Fig. 2F). When plated in appropriate three-dimensional (3D) culture conditions, these MaSCs form solid organoids (Fig. 2F) that are mostly clonal, as judged by analyses of mixtures of MaSCs with or without dTomato expression (fig. S3K). These organoids form rudimentary branches composed of Krt8^+^;Krt14^−^ cells surrounded by Krt14^+^;Krt8^−^ cells, mimicking the complex cellular architecture of the mammary gland (Fig. 2G). Moreover, the Krt14^+^ cells of the outer layer expressed Slug and displayed primary cilia (Fig. 2G), similar to the human and mouse mammary glands in vivo (Fig. 1A and fig. S1A).

We tested the impact of SU5402 in this 3D assay at various concentrations. Consistent with published data (*23*), we found that SU5402 suppresses organoid formation in a dose-dependent manner (fig. S3L), confirming the importance of FGF signaling for stemness. At 20 μM, organoid formation was abolished, while at 10 μM organoid formation was repressed but smaller organoids were able to grow (fig. S3, L and M). The small organoids retained the appropriate architecture, including Krt14^+^ and Slug^+^ cells forming the outer layer of the organoids, but they showed a significant reduction in the fraction of ciliated cells as well as cilium length (Fig. 2H and fig. S2N). Together, these data showed that EMT-TFs activate the expression of ciliogenesis inducers, including *FGFR1*, which promote primary ciliogenesis in MaSC-enriched basal cells of the mammary epithelium, and establish that FGF signaling is critical for MaSC stemness and organoid formation.

### Primary cilia control ubiquitination-dependent GLIS2 inactivation

**Fig. 3.**
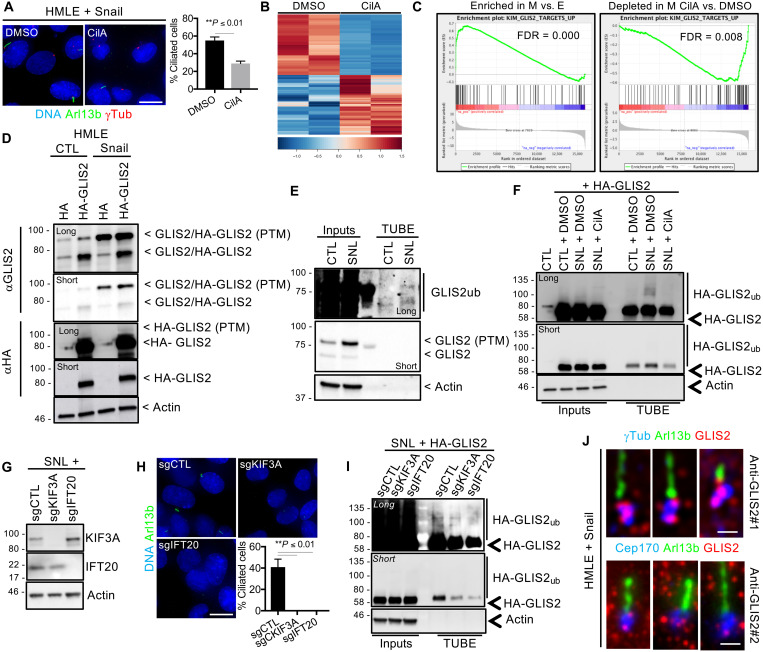
Primary cilia control GLIS2 inactivation. (**A** and **B**) CTL and Snail-expressing HMLE cells were grown until high confluence and serum-starved for 24 hours with DMSO and ciliobrevin A (CilA). (A) Cells were stained for the indicated proteins, and the percentage of ciliated cells was determined. Scale bar, 15 μm (*n* = 3, means ± SEM). Representative results from three independent experiments are shown. (B) Gene expression was analyzed by RNA-seq in both conditions. Heatmaps showing the differentially expressed genes (*q* ≤ 0.05, fold change ≥2) between samples are displayed. (**C**) A GSEA analysis shows significant enrichment of GLIS2 target genes in the list of up-regulated genes in Snail-expressing cells (M) relative to CTL (E) cells (left panel) and depletion in the list of Snail-expressing (M) CilA-treated relative to DMSO-treated cells (right panel). FDR, false discovery rate. (**D**) CTL and Snail-expressing HMLE cells that express HA or HA-GLIS2 were grown as described in (A). GLIS2 and HA-GLIS2 protein expression was analyzed by Western blot. Representative results from three independent experiments are shown. (**E**) CTL- and Snail (SNL)-expressing HMLE cells were grown as described above. Ubiquitinated proteins were purified by TUBE pull-down and analyzed by Western blot. Representative results from three independent experiments are shown. (**F**) CTL- and Snail-expressing HMLE cells that express HA-GLIS2 were grown and treated with DMSO or CilA as described in (A). The impact on protein ubiquitination was analyzed as described in (E). (**G**) *KIF3A* and *IFT20* KOs were validated by Western blot of extracts from the indicated cells. (**H**) The impact on primary ciliogenesis was assessed as described in (A). Scale bar, 15 μm. (**I**) The impact on HA-GLIS2 ubiquitination was analyzed as described in (E). (**J**) GLIS2 localization at the ciliary base in Snail-expressing HMLE cells was determined by immunofluorescence staining of the indicated proteins. Scale bar, 1 μm.

Primary cilia act as cell signaling centers (*24*). To identify signaling pathways that are controlled by primary cilia in mammary epithelial cells, we further expanded our gene expression analysis to Snail-expressing cells grown to high confluence and serum-starved in the presence of DMSO or with ciliobrevin A (CilA), a small-molecule ciliogenesis inhibitor (*25*). As above, replicate samples of cells were stained for Arl13b and γTubulin to assess the presence of primary cilia under the two conditions (Fig. 3A), confirming that ciliogenesis was significantly inhibited by CilA treatment relative to DMSO treatment. Replicate samples were then subjected to RNA-seq (Fig. 3B). This revealed substantial changes in the gene expression profiles of Snail-expressing cells treated with CilA relative to DMSO-treated cells (Fig. 3B).

To identify signaling pathways induced by EMT and primary cilia, we performed gene set enrichment analysis (GSEA). Here, we looked for significantly enriched gene sets that include genes that are up-regulated in Snail-expressing HMLE cells versus CTL cells (Fig. 2A) and are down-regulated in Snail-expressing CilA-treated cells versus DMSO-treated cells (Fig. 3B). This revealed a gene set that is known to be regulated by GLIS2, a transcriptional repressor (Fig. 3C). GLIS2 target genes are up-regulated in the Snail-expressing cells and repressed upon CilA treatment, suggesting that GLIS2 is inactivated in a cilium-dependent manner.

The GLIS2 transcriptional repressor was discovered to be essential for normal development of both the kidney and incisor tooth epithelium (*26*–*28*). Recent studies showed that it acts as a central regulator of stem and progenitor cell maintenance and differentiation (*28*, *29*). Existing data already suggested a link between GLIS2 and primary cilia. First, GLIS2 loss causes nephronophthisis, a renal ciliopathy (*26*). Second, in the kidney and incisor tooth epithelial cells, GLIS2 localizes to primary cilia (*26*, *28*). However, the molecular basis of the interplay between GLIS2 and primary cilia is unknown. Moreover, GLIS2 has not been previously implicated in mammary gland biology. Our sequencing data suggest that primary cilia mediate GLIS2 inhibition in the mammary epithelium.

To explore the underlying mechanism of this repression, we first analyzed *GLIS2* RNA expression in CTL, Snail, Snail + DMSO, and Snail + CilA HMLE variants using our RNA-seq dataset as well as by RT-qPCR analysis. Neither the RNA-seq analysis nor the RT-qPCR analysis indicated that *GLIS2* RNA expression is repressed upon EMT and ciliogenesis (fig. S4, A and B). Notably, two of the handful of reported GLIS2 target genes, *GLI1* and *CDH11 (**26**)*, were up-regulated in Snail versus CTL cells and down-regulated in Snail + CilA versus Snail + DMSO cells (fig. S4B). Thus, we concluded that GLIS2 is inhibited posttranscriptionally.

To assess this possibility, we first introduced hemagglutinin (HA)-tagged GLIS2 or a HA-only control, into HMLE CTL and Snail-expressing cells by viral transduction, and examined its levels by Western blotting with the anti-HA antibody (Fig. 3D). The levels of HA-GLIS2 were similar in both contexts (Fig. 3D, α-HA, short exposure), but a higher molecular weight species, which we presumed represents a posttranslationally modified form of GLIS2, was observed at higher abundance in the Snail-expressing cells (Fig. 3D, α-HA, long exposure). We then extended this analysis to consider the endogenous GLIS2 protein. We reran the same cell extracts, with better separation, and now probed with an anti-GLIS2 antibody (Fig. 3D, α-GLIS2). This detected two major GLIS2 species in both the HA-tagged GLIS2 and the HA control samples from HMLE CTL and Snail-expressing cells (Fig. 3D, α-GLIS2). The higher molecular weight GLIS2 species was present at higher levels in the Snail-expressing cells (Fig. 3D, α-GLIS2). Thus, we conclude that endogenous GLIS2 is subject to posttranslational modifications, and these are up-regulated in the Snail-expressing cells where GLIS2 is inactivated.

Previous studies have reported that GLIS2 can undergo a nondegradative form of polyubiquitination, which results in its inactivation (*30*). Thus, we hypothesized that the higher molecular weight forms of GLIS2 represent polyubiquitinated GLIS2. To address this, we subjected whole-cell lysates from CTL or Snail-expressing HMLE cells to affinity chromatography using tandem ubiquitin-binding entities (TUBEs). Total ubiquitinated proteins were recovered at comparable levels from the two HMLE variants (fig. S4C), but the higher molecular weight GLIS2 species was detected in the Snail-expressing cells but not in the control HMLE cells (Fig. 3E). We therefore conclude that GLIS2 becomes polyubiquitinated as a consequence of EMT induction in HMLE + Snail cells.

A recent large-scale analysis of the ubiquitinome established that GLIS2 can undergo ubiquitination on lysine-251 in liver and leukemic cancer cell lines (*31*). We wondered whether this site of modification plays a regulatory role in the human mammary epithelial cells under study. To test this hypothesis, we expressed wild-type HA-GLIS2, or a mutant variant in which lysine-251 was replaced by an arginine (K251R), in HMLE + Snail cells. Whole-cell lysates were subjected to TUBE, and the purified ubiquitinated proteins were analyzed by Western blot (fig. S4D). While the levels of unmodified wild type and HA-GLIS2_K251R_ were comparable, polyubiquitinated HA-GLIS2_K251R_ was recovered at lower levels than polyubiquitinated wild-type HA-GLIS2, but certainly not eliminated (fig. S4D). This result strongly suggests that GLIS2 is ubiquitinated on K251R, but additional sites of ubiquitination exist. We reasoned that if cilium-dependent ubiquitination enables GLIS2 inactivation and K251 is an important regulatory site, the GLIS2_K251R_ mutant form of GLIS2 would be expected to bypass ciliary inhibition and behave as a constitutively active transcriptional repressor in HMLE + Snail cells. To test this hypothesis, we extracted RNA from HMLE cells that expressed either wild-type or mutant HA-GLIS2 and performed RT-qPCR experiments (fig. S4E). Consistent with this hypothesis, we found that GLIS2_K51R_ mutant significantly repressed *GLI1* and *CDH11* expression, two reported GLIS2 targets, compared to the wild-type GLIS2 species (fig. S4E).

In parallel experiments, we also examined the role of primary cilia in GLIS2 ubiquitination. First, CTL and Snail-expressing cells with wild-type HA-GLIS2 were treated with DMSO or the ciliogenesis inhibitor CilA, and then ubiquitinated proteins were recovered by TUBE. The CTL + DMSO, Snail + DMSO, and Snail + CilA samples all yielded comparable levels of total ubiquitinated proteins and total HA-GLIS2 protein (Fig. 3F and fig. S4F). Nevertheless, polyubiquitinated HA-GLIS2 was up-regulated in the Snail + DMSO cells compared to the CTL + DMSO cells, and this was abolished in the Snail + CilA cells (Fig. 3F), strongly suggesting that these are cilium-dependent events.

As an alternative approach, we also examined ubiquitination of HA-GLIS2 in Snail-expressing cells that had CRISPR KO of *KIF3A* and *IFT20*, two essential ciliogenesis regulators. For this, we selected five CRISPR single-cell clones that displayed complete KIF3A or IFT20 protein loss and then pooled them to minimize effects of clonal variation (Fig. 3G). Control pools of wild-type single-cell clones were also generated. We observed that ciliogenesis was abolished in the KIF3A and IFT20 KO cells but not in the wild-type controls (Fig. 3H), and this decreased the levels and pattern of polyubiquitinated HA-GLIS2 species (Fig. 3I) in a comparable manner to the pharmacological inhibition of ciliogenesis (Fig. 3F). Together, our ubiquitination analyses argue that primary cilia promote polyubiquitination of GLIS2 in mammary epithelial cells, on its K251 residue and other sites, and that this enables GLIS2 inactivation.

GLIS2 has been reported to interact with, and be regulated by, different members of the Bardet-Biedl syndrome (BBS) family of proteins (*30*, *32*). These proteins are important regulators of ciliary signaling and are enriched at the ciliary base within ciliary vesicles (*33*). Given this, we wondered whether GLIS2 might be recruited to this region in HMLE Snail-expressing cells that assemble primary cilia. We addressed this by costaining HMLE Snail-expressing cells for GLIS2 and for markers of primary cilia, Arl13b and γTubulin (Fig. 3J). This detected endogenous GLIS2 at the base of the cilium in vesicle-like structures. It seems likely that this localization contributes to the mechanism of inhibition of GLIS2 by primary cilia.

### GLIS2 is a central regulator of basal MaSC and mammary gland development

Our previously reported data established that EMT-induced primary ciliogenesis in MaSC-enriched basal cells promotes their stemness (*11*). Given this finding, we undertook to test the role of the primary cilium–GLIS2 signaling axis in this process. We initiated this study in our HMLE model, in which the ability to form mammospheres, which are 3D floating structures composed of undifferentiated cells, serves as a measure of their SC-like properties (*2*).

**Fig. 4.**
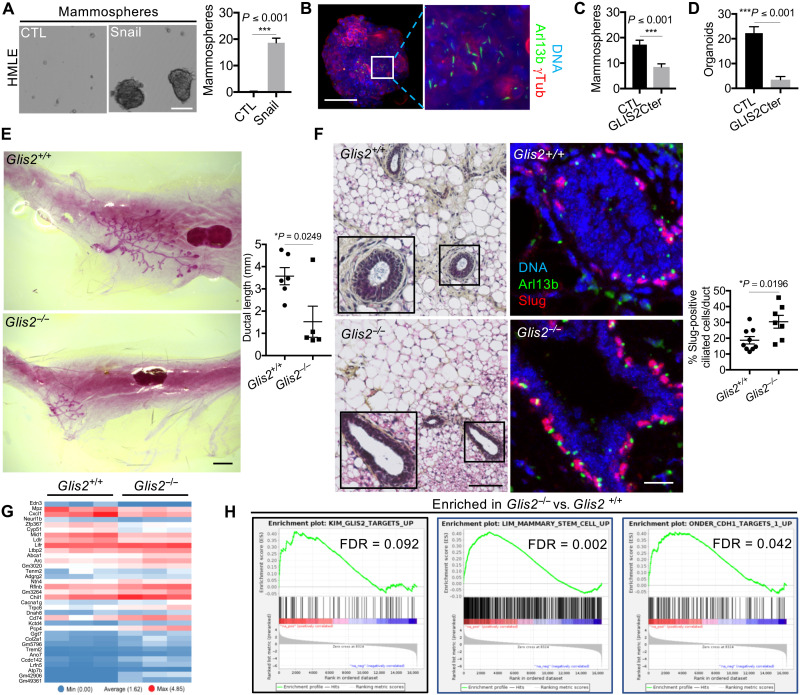
GLIS2 is a central regulator of basal MaSC and mammary gland development. (**A** to **C**) Mammospheres from Snail-expressing HMLE cells were examined for morphology by brightfield microscopy or for ciliated cells by immunofluorescence for the indicated proteins by light sheet microscopy. Mammosphere-forming capacity was quantified for the indicated cells (*n* = 3, means ± SEM). Representative results from three independent experiments are shown. Scale bars, 100 μm. (**D**) Organoid-forming capacity was determined for sorted MaSC-enriched basal cells in which GLIS2Cter was overexpressed (*n* = 3, means ± SEM). Representative results from three independent experiments are shown. (**E**) Mammary glands from *Glis2*^+/+^ or *Glis2*^−/−^ female mice (4 weeks old, *n* ≥ 5) were stained with carmine alum, and ductal length was analyzed after whole-mount preparation. Scale bar, 1 mm. (**F**) Paraffin sections from the mammary glands were stained with H&E or for the indicated protein. The percentage of Slug^+^ ciliated cells was quantified for each genotype (*n* = 3, means ± SEM). Representative images are shown. Scale bars, 100 μm (H&E; inset: 2×) and 50 μm (immunofluorescence). (**G**) Gene expression was analyzed by RNA-seq in *Glis2^+/+^* and *Glis2^−/−^* mammary cells (three mice per genotype). Heatmap showing the differentially expressed genes (*q* ≤ 0.05, fold change ≥1.5) between samples is displayed. (**H**) A GSEA analysis shows significant enrichment of GLIS2 target genes, and of genes that mark EMT and MaSCs, in the list of up-regulated genes in *Glis2^−/−^* compared to *Glis2^+/+^* cells.

To begin, we assessed primary cilia representation in mammospheres arising from HMLE cells that have gained the ability to form these structures upon Snail expression, doing so by staining for Arl13b and γTubulin (Fig. 4, A and B). This showed that the undifferentiated cells in mammospheres formed primary cilia (Fig. 4B). To investigate the role of GLIS2 in this process, we took advantage of a previously described GLIS2 truncation mutant (*GLIS2* c. 1_444del), termed GLIS2Cter, which can act as a constitutive transcriptional repressor (*34*). Thus, we introduced GLIS2Cter or a control vector into our Snail-expressing HMLE cells (fig. S5A). We first confirmed the ability of GLIS2Cter to mediate transcriptional repression using qRT-qPCR to gauge the expression of the reported GLIS2 target genes, *GLI1* and *CDH11*, and found that both transcripts were down-regulated, relative to the control vector cells (fig. S5B). Consistent with the notion that GLIS2 is downstream of EMT-TFs and the induction of primary cilia, GLIS2Cter did not alter the M-like status of these cells, as judged by morphology and expression of E-M markers (fig. S5, A and C), and their ability to form primary cilia (fig. S5D). In addition, GLIS2Cter did not alter the 2D proliferation rate of Snail-expressing cells (fig. S5E). In subsequent mammosphere assays, we found that GLIS2Cter expression significantly inhibited the mammosphere-forming capacity of the Snail-expressing HMLE cells relative to control vector cells (Fig. 4C).

Given these findings, we extended our analyses to primary basal MaSCs. Specifically, we isolated mouse basal MaSCs by FACS, as described above, and generated populations with ectopic expression of either GLIS2Cter or control vector. We then examined the ability of these cells to generate organoids and observed a significant reduction in response to GLIS2Cter expression (Fig. 4D). Together, these data showed that perturbation of the primary cilium–GLIS2 axis results in inhibition of stem cell–like cells in both the HMLE population and the basal murine MaSCs and thus impairs ex vivo organogenesis.

We reasoned that if Glis2 were important for regulation of MaSCs in vivo, then its deletion should perturb mammary gland development in mice carrying germline KO of the *Glis2* gene. We further hypothesized that the resulting mammary defects would reflect inappropriate expansion of MaSCs at the expense of cell differentiation and proper lineage commitment. To test these predictions, we examined mammary gland development in 4-week-old *Glis2*^+/+^ and *Glis2*^−/−^ female mice through carmine alum staining of whole-mount mammary glands. In agreement with our hypothesis, ductal morphogenesis was profoundly impaired in *Glis2*-deficient mice, as evidenced by the presence of only small mammary rudiments relative to the *Glis2*^+/+^ controls (Fig. 4E). In addition, hematoxylin and eosin (H&E) staining of paraffin sections showed that *Glis2*^+/+^ glands had the expected bilayered epithelium, but this organization was disrupted in *Glis2*^−/−^ mice (Fig. 4F). *Glis2*^−/−^ rudimentary ducts were enriched for Slug^+^ ciliated cells compared to *Glis2^+/+^* ducts (Fig. 4F), and cells expressing TSPAN8, a plasma membrane protein that is expressed in basal MaSCs and, to a lesser extent, in luminal progenitor cells (fig. S5F) (*35*). Collectively, these data support our hypothesis that GLIS2 deficiency results in expansion of MaSCs.

To further explore the molecular consequences of *Glis2* deletion, we isolated mammary epithelial cells from *Glis2*^+/+^ and *Glis2^−/−^* mice by FACS and conducted RNA-seq (Fig. 4G and fig. S5G). This revealed changes in gene expression between mammary epithelial cell variants (Fig. 4G). We then performed GSEA to identify gene sets that were significantly different between the *Glis2^−/−^* and *Glis2^+/+^* populations (Fig. 4H). As expected, we found that the GLIS2 target gene set was significantly enriched in the *Glis2^−/−^* cells, consistent with the loss of GLIS2 repressor function (Fig. 4H). Moreover, the top scoring gene sets included MaSC and EMT signatures (Fig. 4H), which was entirely consistent with ectopic expansion of MaSCs in the mammary gland of Glis2-null mice. We also found that gene sets associated with hyperactivation of known MaSC signaling pathways, Notch and Wnt/β-catenin (fig. S5H). This is particularly gratifying, as published work showed that GLIS2 negatively regulates the Wnt/β-catenin signaling pathway by directly binding β-catenin and inhibiting its transcriptional activity (*36*). Collectively, our data showed that GLIS2 controls the expansion of basal MaSCs in a manner that is required for normal mammary gland development, and raises the possibility that this occurs via regulation of Wnt/β-catenin signaling.

### Primary cilium–dependent regulation of GLIS2 controls claudin-low MaTIC stemness and tumorigenicity

**Fig. 5.**
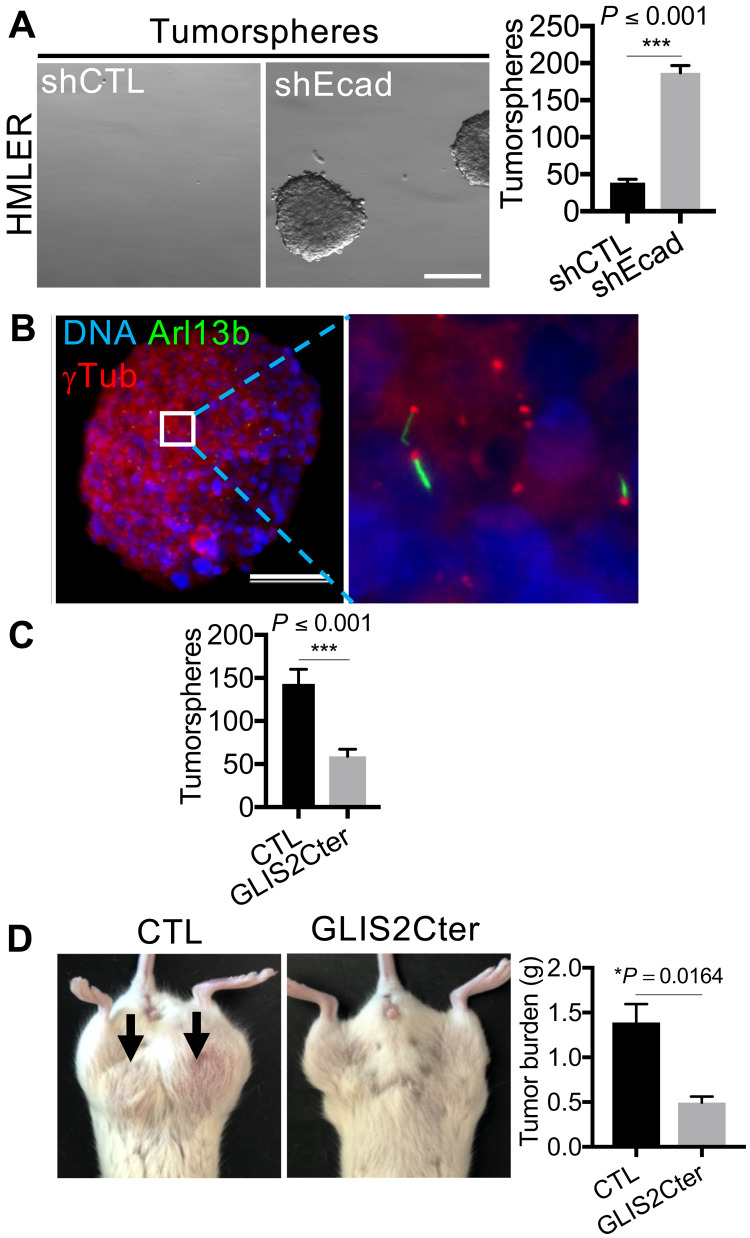
The primary cilium–GLIS2 axis controls claudin-low MaTIC stemness and tumorigenicity. (**A** to **C**) Tumorspheres from shEcad-expressing HMLER cells were examined for morphology by brightfield microscopy or for ciliated cells by immunofluorescence for the indicated proteins and light sheet microscopy. Tumorsphere-forming capacity was quantified for the indicated cell variants (*n* = 3, means ± SEM). Representative results from three independent experiments are shown. Scale bars, 300 μm (brightfield image) and 100 μm (immunofluorescence image). (**D**) Bilateral orthotopic implantations were conducted with HMLER shEcad CTL or GLIS2Cter-expressing cells. Representative mice are shown. Tumor burden per mouse (means ± SEM) was determined 8 weeks after implantation, with two sites of implantation per mouse and four mice per cell type.

We have previously shown that the role of primary cilia is not only important for normal basal MaSCs but also essential for the tumor-initiating capacity of MaTICs in an orthotopic murine carcinoma model (*11*). This analysis was conducted using HMLE cells transformed with H-RASG12V (called HMLER cells), which, upon E-cadherin knockdown, become more M-like (fig. S6A) and acquire MaTIC properties (*11*). These cells can form tumorspheres composed of nondifferentiated cells that display cilia in vitro (Fig. 5, A and B) and generate ciliated tumors that display the hallmarks of claudin-low breast cancers (fig. S6B).

We therefore asked whether primary cilia control GLIS2 in these transformed HMLER cells. We began by assessing the cilium-dependent gene expression events in HMLER variants. For this, we grew HMLER shCTL and shEcadherin (shEcad)–expressing cells in ciliogenesis-permissive conditions, with or without DMSO and CilA. For each condition, samples were stained for Arl13b and E-cadherin to assess primary cilia representation. These analyses confirmed that E-cadherin loss in HMLER cells led to a marked increase in the percentage of ciliated cells, relative to shCTL cells (fig. S6C), and that this ciliogenesis was reduced significantly by treatment with CilA (fig. S6C). Parallel samples were analyzed by RNA-seq, revealing substantial changes in gene expression profiles between these HMLER populations (fig. S6D). GSEA revealed that the GLIS2 target genes were up-regulated in shEcad-expressing versus shCTL cells and down-regulated in shEcad-expressing CilA versus DMSO-treated cells (fig. S6E). Thus, GLIS2 is inhibited in a cilium-dependent manner in transformed HMLER cells enriched for MaTICs in a similar manner to that observed in their nontransformed HMLE counterparts.

We next asked whether GLIS2 activity influences the properties of these HMLER cells by generating HMLER shEcad populations expressing GLIS2Cter. As with Snail-expressing HMLE cells, this perturbation did not alter the E-M status, primary ciliogenesis, or proliferation rate of the HMLER + shEcad cells in monolayer culture (fig. S6, F to I). However, GLIS2Cter did repress *GLI1* and *CDH11* gene expression (fig. S6J), validating its activity. GLIS2Cter expression decreased the tumorsphere-forming capacity of HMLER shEcad cells relative to the control vector cells (Fig. 5C).

To further validate the key role of GLIS2 in tumors, we also compared the tumorigenic capacity of GLIS2Cter-expressing and CTL HMLER shEcad cells in orthotopic tumor implantation experiments. Consistent with our in vitro assays, the GLIS2Cter expression significantly reduced the in vivo tumorigenic capacity of the HMLER shEcad cells (Fig. 5D), in a similar manner to mutation of *KIF3A*, which impairs primary ciliogenesis (fig. S6, K to M). These data show that the primary cilium–dependent regulation of GLIS2 is required for the proliferative and tumorigenic capacities of MaTICs, which form tumors displaying hallmarks of claudin-low breast cancers.

**Fig. 6.**
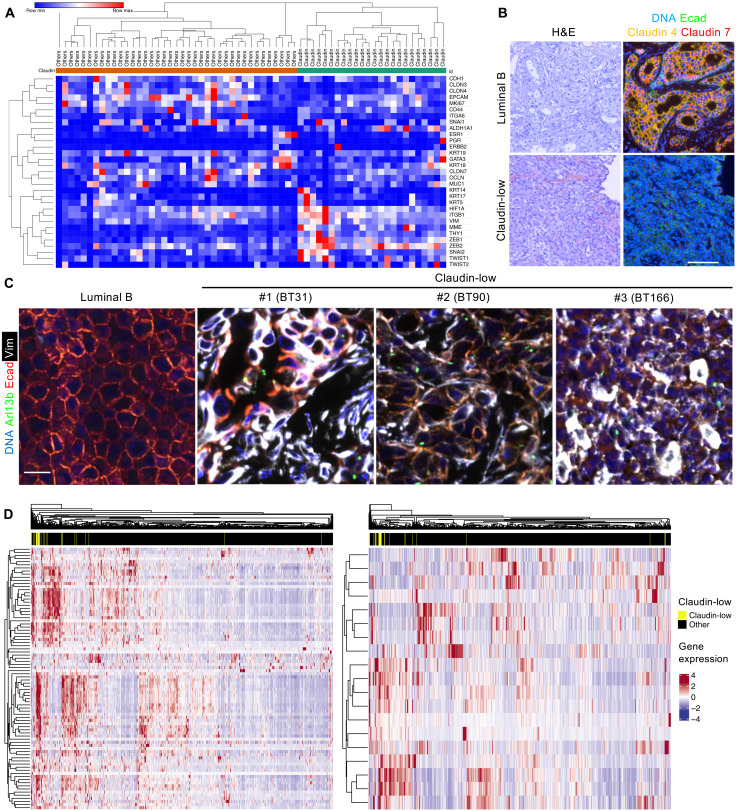
Primary cilia mark claudin-low tumor cells in E-M states, and high expression of GLIS2 target genes is a hallmark of claudin-low tumors. (**A**) Human breast tumor fragments from 62 independent patients were flash-frozen after surgery. RNA was extracted from sections of frozen tumors, and gene expression was analyzed by RNA-seq. Expression of a subset of genes was analyzed to discriminate claudin-low tumors from other molecular subtypes. The heatmap showing the differential expression of the genes is displayed. (**B** and **C**) Paraffin sections of breast tumors were stained with H&E or for the indicated proteins. Scale bars, 100 μm (B) and 20 μm (C). (**D**) Heatmaps showing the differential expression of GLIS2 target genes (left panel), and the reference list of genes used to mark claudin-low tumors (right panel), in breast cancers are displayed, along with their ability to segregate claudin-low tumors from other breast tumors.

To further analyze primary ciliogenesis and GLIS2 in claudin-low human breast cancers, we established a tumor biobank by characterizing tumor samples in a previously assembled TNBC biobank. We extracted total RNA from sections of frozen tumors (*n* = 62) and analyzed gene expression by RNA-seq. Among the 62 TNBCs, 23 claudin-low tumors were then identified on the basis of their differential expression of a panel of genes (Fig. 6A), previously identified by Prat and colleagues (*37*). We were able to stain paraffin sections for 20 of the claudin-low tumors, along with three luminal B tumors (as controls) for H&E and also for E-cadherin, claudin 4, and claudin 7 (Fig. 6B). We found that the claudin-low tumors displayed a poorly differentiated phenotype, in contrast to the luminal B tumors, which all demonstrated the typical appearance of differentiated breast cancers (Fig. 6B). The luminal tumors expressed higher levels of epithelial markers than did 85% of the claudin-low tumors (Fig. 6B and fig. S7A). We also costained luminal and claudin-low tumor sections for vimentin, E-cadherin, Arl13b, and γTubulin to assess EMT status and primary cilia representation. We detected ciliated cancer cells that coexpressed E-cadherin and vimentin specifically in claudin-low tumors, but not in luminal tumors, indicating that they reside in an intermediate EMT state (Fig. 6C and fig. S7B). Moreover, these cells represented a small fraction of overall tumor cells (fig. S7C), consistent with the hypothesis that the ciliated claudin-low tumor cells are MaTICs.

We next performed complementary gene expression analysis through deep RNA-seq in a subset of the claudin-low tumors that we identified, as well as in luminal B control tumors (fig. S7D). This revealed substantial differences in gene expression between claudin-low and luminal B tumors (fig. S7D). We performed GSEA analysis to identify gene sets that are associated with claudin-low ciliated tumors. In a similar manner to our analysis of the Glis2-deficient mammary gland, the top scoring gene sets included the GLIS2 and MaSC sets (fig. S7E), as well as gene sets associated with hyperactivation of Notch and Wnt/β-catenin signaling (fig. S7E).

To determine whether high expression of the GLIS2 signature is a hallmark of claudin-low tumors across breast cancers in general, we compared the expression of GLIS2 target genes in 1082 breast cancers, using a public dataset from The Cancer Genome Atlas (TCGA) (*38*). We found that high expression of GLIS2 target genes enables the segregation of claudin-low tumors from other breast tumors in a manner similar to the Prat *et al*. (*37*) reference panel of genes used to mark claudin-low cancers (Fig. 6D). Moreover, GSEA revealed that the GLIS2 gene set is significantly enriched in the signature that was initially used to define the claudin-low tumors (fig. S7F) (*37*). Collectively, our data showed that primary cilia are assembled in claudin-low breast tumor cells that are enriched in MaTICs, in which they control GLIS2 inactivation to promote the tumorigenic capacity of these cells. Furthermore, high expression of GLIS2 target genes serves as a hallmark of claudin-low breast cancers.

## DISCUSSION

Our previously reported data established that EMT programs induce ciliogenesis in mammary epithelial cells (*11*). However, we lacked a mechanistic understanding of the link between EMT and primary ciliogenesis, including the degree to which ciliogenesis is a stable or a transient response upon EMT activation, as well as the underlying molecular mechanisms. Our data reinforce the connection between EMT programs and primary ciliogenesis in both the human and mouse mammary glands. Specifically, we show that the propensity to form primary cilia closely follows entrance into mixed epithelial-mesenchymal phenotypic states and that primary cilia are assembled in mammary epithelial cells that display an array of M-like phenotypes. While we do not see a decrease in the percentage of ciliated cells in the most mesenchymal phenotypic states in our systems, we do not exclude the possibility that primary ciliogenesis is repressed at more extreme mesenchymal EMT states. This latter hypothesis is supported by an elegant study of epithelial-myofibroblast transition (EMyT), a more extreme type of EMT that can occur in kidney epithelial cells (*39*). Here, primary cilia are induced during early transition states, where they promote EMyT progression but are ultimately repressed when the cells acquire the highly mesenchymal, myofibroblast phenotype (*39*). In yet another context, the murine coronary vasculature, a link between EMT and primary ciliogenesis was recently identified during development (*40*). Here, the authors found that epicardial cells that undergo EMT assemble primary cilia, and that genetic disruption of ciliogenesis in these cells perturbed EMT and migration, causing coronary artery developmental defects (*40*). Collectively, these findings cement a strong connection between EMT and primary ciliogenesis in multiple contexts and underscore the concept that EMT is not a simple binary switch between E and M states, but instead generates a spectrum of transitional phenotypic states that can have a distinctive relationship with the primary cilium biology. Our data established that EMT induces primary ciliogenesis upon entry into intermediate transition states in mammary epithelial cells of the human and mouse mammary gland to support stemness.

The precise molecular mechanisms by which EMT programs induce primary ciliogenesis were previously unknown. Our data show that EMT-TFs activate the transcription of ciliogenesis inducers in mammary epithelial cells. Specifically, we found that FGFR1 expression is induced by EMT-TFs upon entry into intermediate EMT states during which FGFR1 is activated. We show that FGFR1 is responsible, at least in part, for primary cilium assembly and elongation upon activation of EMT programs. A recent study found that FGFR1 promotes ciliogenesis in the hair cells of the inner ear of the chick embryo by phosphorylating the protocadherin Pcdh15 (*20*). We found another member of the protocadherin family, *PCDHB15*, together with *FGFR1*, in our identified list of 47 putative ciliogenesis inducers (Fig. 2C), raising the intriguing possibility that FGFR1 cooperates with protocadherin β-15 in promoting primary ciliogenesis in mammary epithelial cells. Of note, FGFR1 cooperates with FGFR2 to promote mammary gland development and regeneration by enabling the stemness of MaSCs (*22*, *23*). Our own observations show that FGFR inhibition represses both ciliogenesis and organoid-forming capacity in MaSCs. Collectively, these data suggest that FGFR1 controls ciliogenesis, thereby enabling basal MaSC stemness and mammary gland development.

Our previous work revealed that primary cilia control Hedgehog signaling in mammary cells (*11*). In the current study, we used gene expression analyses to gain a broader, and unbiased, insight into the signaling pathways that depend on the primary cilium in mammary cells that control their stemness. These analyses revealed that cilia control the central signaling node GLIS2. This was highly gratifying because, although GLIS2 is a relatively understudied protein, it had already been linked to EMT, primary cilia, and stemness in other contexts. With regard to EMT, GLIS2 was found by others to repress EMT in the kidney (*26*). We did not observe evidence of increased mesenchymal-epithelial transition (MET) when GLIS2 was ectopically activated in M-like ciliated mammary cells, leading us to conclude that GLIS2 does not directly repress EMT in this setting. Nevertheless, our in vitro and in vivo studies show that GLIS2 represses expansion of MaSCs that activate an EMT program in the mammary epithelium. With regard to primary cilia, GLIS2 was shown by others to localize to the primary cilium in incisor tooth epithelial stem cells and in kidney cells (*26*, *28*). Our data show that GLIS2 is polyubiquitinated and inhibited in a cilium-dependent fashion in M-like mammary cells. Moreover, it identifies lysine-251 as one, but not the only, residue that is ubiquitinated. Mutation of lysine-251 causes down-regulation of two reported GLIS2 target genes, *GLI1* and *CDH11*, supporting the notion that GLIS2 ubiquitination serves to inhibit GLIS2’s repressor activity. In addition to ubiquitination, we also found that GLIS2 is localized to the base of primary cilia in M-like mammary cells. Notably, the BBS family of proteins localizes to primary cilia, or the ciliary base in a similar manner to GLIS2, and several BBS proteins have been reported to bind to GLIS2 (*30*, *32*, *33*). This includes BBS11, a known E3 ubiquitin ligase (*30*). Thus, we hypothesize that primary cilia serve as cell signaling platforms that recruit GLIS2 and enable its ubiquitination and consequent inactivation (fig. S8), potentially with the involvement of BBS11. Last, our data show that GLIS2 acts to repress stemness in mammary cells, and this is inhibited in MaSCs.

As mentioned, previous reports revealed that GLIS2 controls normal development of both the kidney and the incisor tooth epithelium (*26*–*28*). Our own data show that GLIS2 also plays a critical role in normal mammary gland development, which is consistent with our discovery of its role in MaSC regulation. More specifically, we found a major morphogenetic defect at puberty in *Glis2*-deficient female mice. We detected only rudimentary trees in *Glis2*-deficient animals, in which the normal bilayer structure of the mammary epithelium is disrupted and an excess of basal MaSCs, which activate an EMT program and are ciliated, was detected. These findings suggest that there is an inappropriate persistence or expansion of the SC compartment and/or a failure of engagement of the differentiation program at the appropriate developmental time. The signaling pathway(s) downstream of GLIS2 that control(s) MaSC stemness remain to be fully elucidated, but it is intriguing to note that GLIS2 has been shown to repress two key stem cell signaling pathways, Hedgehog and Wnt (*36*, *41*). This intersects with our previous finding that Hedgehog signaling acts downstream of primary ciliogenesis in basal MaSCs and MaTICs (*11*), and our gene expression analyses in *Glis2*-deficient mammary epithelial cells and claudin-low tumors, which suggest that Wnt signaling may be directly downstream of GLIS2 in controlling MaSC and MaTIC stemness.

Our previous work revealed a critical role for primary cilia in MaTICs of the more mesenchymal HMLER cell line, which yield tumors that display the hallmarks of claudin-low human breast cancers (*11*). Here, we found that primary cilia control GLIS2 in these MaTICs, thereby enabling their self-renewal and tumorigenic capacities both in vitro and in vivo. To directly address the relevance of these findings in human breast tumors, we established a biobank of claudin-low breast tumor samples. Subsequently, we found that claudin-low breast tumors are enriched in tumor cells that display an M-like phenotype, assemble primary cilia, and express high levels of GLIS2 target genes, as well as MaSC and Wnt signaling gene programs. Notably, we found that a GLIS2 signature identifies claudin-low tumors from other breast cancer subtypes as effectively as the previously identified claudin-low signature. In conclusion, by establishing molecular mechanisms by which EMT induces ciliogenesis and thereby controls MaSC and MaTIC biology, our findings reveal a novel aspect of the cell biology critical to the development of the mammary gland and the formation of claudin-low breast cancers.

## MATERIALS AND METHODS

### Animals

Transgenic and wild-type mice were housed and handled in accordance with protocols approved by the Animal Care Committees of the Massachusetts Institute of Technology (USA), the National Institutes of Health (USA), the University of Iowa (USA), and the University of Rennes (France). Slug-IRES-YPF and Glis2 mutant mice were generated and selected as previously described (*3*, *27*). C57BL/6J and nonobese diabetic severe combined immunodeficiency (NOD SCID) animals were obtained from the Jackson Laboratory (stock numbers 000664 and 001303).

### Human samples

Patient reduction mammoplasty tissue samples and breast tumor samples were obtained in compliance with all relevant laws. Breast cancer patients (*n* = 62) were diagnosed and treated at the Centre Eugène Marquis between 2013 and 2018; none received chemotherapy, endocrine therapy, or radiation therapy before surgery. Tissues were collected by a pathologist after resection by a surgeon. Some fragments were paraffin-embedded (normal tissues and tumors), other fragments were flash-frozen (tumors), and other fragments were dissociated within 2 hours after surgical resection (tumors). Briefly, breast tumor pieces were weighed and cut into small fragments (<2 mm^3^), which were then dissociated with a tumor dissociation kit (human) using a mechanical-chemical-mechanical cycle in gentleMACS Dissociator (Miltenyi Biotec), according to the manufacturer’s recommendations. Next, a cycle of mechanical-chemical-mechanical dissociation was performed, and dissociated cells were resuspended in RPMI 1640. Macroscopic pieces were removed using a Corning cell strainer (70 μm). Tumor cells were then washed twice in RPMI 1640 (20 ml) and counted using a hemocytometer. Viable cells were then cryopreserved.

### Primary mouse mammary epithelial cell isolation and FACS

Mammary glands from 10- to 16-week-old C57BL/6J females were minced and dissociated using collagenase/hyaluronidase (STEMCELL Technologies) diluted (1:10) in Dulbecco’s modified Eagle medium (DMEM)/F-12 medium at 37°C for 5 hours under constant agitation. Mammary glands from 4-week-old Glis2 wild-type or mutant females were dissociated using collagenase/hyaluronidase (STEMCELL Technologies) diluted (1:10) in DMEM/F-12 medium overnight at room temperature. The dissociated glands were spun down at 450*g* for 5 min and resuspended in ammonium chloride solution (STEMCELL Technologies) diluted (4:1) in Hanks’ balanced salt solution buffer supplemented with 10 mM Hepes and 2% fetal bovine serum (FBS) (HF buffer). The samples were spun down and resuspended in warm trypsin-EDTA (STEMCELL Technologies) by pipetting for 3 min. Trypsin was inactivated by addition of HF buffer. The digested samples were spun down and further digested with dispase solution (STEMCELL Technologies) supplemented with deoxyribonuclease I (0.1 mg/ml) for 1 min. Cell suspensions were diluted with HF buffer and filtered through a 40-μm cell strainer to collect single cells. To separate various cell populations, single cells were stained with a Live-Dead fixable violet dye and antibodies against TER-119, CD31, CD45 [phycoerythrin (PE)/Cy7; BioLegend], CD24 (PE; BioLegend), and CD49f [allophycocyanin (APC); BioLegend]. Stained cells were sorted on BD FACSAria II and FACS Fusion sorters.

### 2D cell culture and treatments

Primary mammary epithelial cells were cultured in EpiCult-B mouse medium (STEMCELL Technologies) supplemented with proliferation supplements, recombinant human epidermal growth factor (EGF; 10 ng/ml), recombinant human basic fibroblast growth factor (10 ng/ml), heparin (4 μg/ml), and penicillin/streptomycin. HMLE cells were cultured in a 1:1 mixture of DMEM/F-12 supplemented with 10% FBS, insulin (0.01 mg/ml), hydrocortisone (0.48 μg/ml), and complete mammary epithelial cell growth medium (MEGM) supplemented with bovine pituitary hormone (Lonza). For activating tetracycline-inducible Snail and Zeb1 expression, cells were grown with doxycycline hyclate (1 μg/ml; Sigma-Aldrich) in medium. For all ciliogenesis assays, cells were grown until high confluence and DMEM/F12 was used to serum starve the cells. For FGFR1 inhibition, cells were treated with SU5402 (Sigma-Aldrich) in DMEM/F12 medium.

### Mammary organoid culture

Matrigel organoid culture was performed as described previously (*11*). Briefly, freshly isolated mammary epithelial cells or transduced cells were cultured in complete EpiCult-B medium (STEMCELL Technologies) containing 5% Matrigel (Corning), 5% heat-inactivated FBS, EGF (10 ng/ml), FGF (10 ng/ml), heparin (4 μg/ml), and 5 μM Y-27632. Cells were seeded at 2000 cells per well in 96-well ultralow attachment plates (Corning). Organoids were counted 7 to 14 days after seeding. For FGFR1 inhibition, SU5402 was added to the medium at 10 μM.

### Mammosphere and tumorsphere culture

Cells were cultured in complete MammoCult medium (STEMCELL Technologies) containing heparin (4 μg/ml), hydrocortisone (0.48 μg/ml), and 1% methyl cellulose. Nontransformed cells were seeded at 1000 cells per well and transformed cells at 150 cells per well in 96-well ultralow attachment plates (Corning). Spheres were counted 7 to 14 days after seeding.

### Mammary gland whole-mount analysis

Glands were fixed in Carnoy’s fixative (100% EtOH, chloroform, and glacial acetic acid; 6:3:1) for 4 hours, washed in 70% ethanol for 15 min and gradually rehydrated in 70, 35, and 15% ethanol baths for 5 min each, and stained with carmine alum overnight at room temperature. Glands were washed with sequential 5-min washes in 50, 70, 95, and 100% ethanol; cleared in xylene overnight; and stored in methyl salicylate until analysis using a Nikon macroscope. Images were processed with ImageJ.

### Tumorigenesis assay

For orthotopic cell implantations, tumor cells were resuspended in a 1:1 mixture of complete MEGM medium with Matrigel. Cells in 20 μl were injected bilaterally into inguinal mammary fat pads of 8-week-old NOD SCID females. Tumors were resected 8 weeks after implantation, and their mass was determined to establish the tumor burden per animal.

### Immunohistochemistry, immunofluorescence, and image analysis

Five-micrometer paraffin tissue sections of formalin-fixed mammary glands and breast tumors were stained following the RUO DISCOVERY Universal staining procedure (Roche) using a Discovery ULTRA staining module. Cells fixed in 4% paraformaldehyde for 10 min on glass coverslips were permeabilized with 0.3% Triton X-100 for 10 min and blocked with 5% goat serum for 1 hour before staining for 1 hour with primary antibodies. Organoids and spheres fixed in 4% paraformaldehyde for 30 min were permeabilized with 0.3% Triton X-100 for 30 min and blocked with 5% goat serum for 1.5 hours before staining overnight at 4°C with primary antibodies. The following primary antibodies were used: Arl13b (1:100; NeuroMab 73-287), YFP (1:100; Cell Signaling Technology 2956), Slug (1:100; Cell Signaling Technology 9585), acetylated tubulin (1:500; Cell Signaling Technology 5335), γ-tubulin (1:50; Sigma-Aldrich T5326), smooth muscle actin (1:100; Abcam ab21027), E-cadherin (1:200; Cell Signaling Technology 3195), Cep170 (1:200; Thermo Fisher Scientific 10383523), Krt8 (1:1000; DSHB TROMA-I-s), Krt14 (1:1000; BioLegend 905301), claudin 4 (1:200; Thermo Fisher Scientific 10303233), claudin 7 (1:200; Thermo Fisher Scientific 10537403), large T (1:200; Santa Cruz Biotechnology sc-20800), VIM (1:200; Dako M0725), GLIS2 (1:100; Aviva ARP30037), GLIS2 (1:100; Abcam ab238589), and TSPAN8 (1:500; gift from J. Visvader). The following secondary antibodies were used: anti-mouse 488 (1:500; Life Technologies A11001), anti-mouse 546 (1:500; Life Technologies A11003), anti-mouse immunoglobulin G1 (IgG1) 647 (1:500; Life Technologies A21240), anti-mouse IgG2A 488 (1:500; Life Technologies A21131), anti-rabbit 488 (1:500; Life Technologies A21206), anti-rabbit 546 (1:500; Life Technologies A11010), anti-rabbit 647 (1:500; Life Technologies A31573), and anti-rat 568 (1:500; Life Technologies A11077). Mounted coverslips with cells were examined using DeltaVision Olympus IX71 microscopes. Z-stacks were deconvolved (Softworx) and processed with ImageJ. Organoids and spheres were embedded in low–melting point agarose and analyzed using a Lightsheet Z1 Zeiss microscope. Z-stacks were analyzed with Imaris and ImageJ.

### TUBE assay

HMLE variants were washed with phosphate-buffered saline (PBS) before protein extraction with lysis buffer [PBS, protease inhibitor cocktail (Sigma-Aldrich), phosphatase inhibitor cocktail (Sigma-Aldrich), 0.1% NP-40, and 100 μM PR-619]. The pull-down of ubiquitinated proteins from protein extracts was performed with TUBE 2 agarose beads (LifeSensors), following the manufacturer’s instructions. Briefly, protein extracts were incubated for 30 min on uncoupled agarose to remove nonspecific binding and 2 hours on equilibrated TUBE 2 agarose on a rotating wheel. Beads were washed three times with lysis buffer. Proteins were heat-denatured and eluted in Laemmli buffer and analyzed by Western blot.

### Western blot experiments and cell cycle analysis

These experiments were conducted using standard procedures as described previously (*42*, *43*). Western blots were performed using the primary antibodies against Snail (1:500; Cell Signaling Technology 3879), Twist (1:200; Abcam ab50887), Zeb1 (1:300; Santa Cruz Biotechnology sc-25388), E-cadherin (1:1000; Cell Signaling Technology 3195), vimentin (1:1000; Dako M0725), fibronectin (1:1000; BD Biosciences 610077), N-cadherin (1:1000; BD Biosciences 610920), KIF3A (1:1000; ProteinTech 13930-1- AP), IFT20 (1:100; ProteinTech 13615-1-AP), FGFR1 (1:1000; Abcam ab76464), pFGFR1 (1:500; Cell Signaling 3471), BBS11 (1:1000; ProteinTech 10326-1-AP), HA (1:1000; Roche 11867423001), red fluorescent protein (1:1000; Rockland 600-401-379S), AKT (1:1000; Cell Signaling Technology 9272), pAKT (1:1000; Cell Signaling Technology 4060), MEK (1:1000; Cell Signaling Technology 9122), pMEK (1:1000; Cell Signaling Technology 9121), Ub (P4D1) (1:300; Santa Cruz Biotechnology sc-8017), GLIS2 (1:100; Aviva ARP30037), HSP90 (1:1000; BD Biosciences 610418), and actin (1:1000; Sigma-Aldrich 20-33) and secondary antibodies horseradish peroxidase–coupled anti-mouse or anti-rabbit (1:5000; GE Healthcare and Jackson Laboratory). The cell cycle analysis was performed using HMLE cells that were fixed in 70% ethanol for 1 hour on ice. DNA was stained with propidium iodide/ribonuclease staining buffer (BD Biosciences) for 15 min. DNA content of 30,000 cells for each condition was determined using a BD Fortessa X20 flow cytometer and the FlowJo software.

### Quantitative RT-PCR

Total RNA was isolated using the PicoPure RNA Isolation Kit (Thermo Fisher Scientific) for mouse cells or the RNeasy Kit (Qiagen) for human cells, and complementary DNAs (cDNAs) were generated with random primers and SuperScript III Reverse Transcriptase (Life Technologies). RT-qPCR reactions were performed with the StepOnePlus RT-PCR System (Life Technologies) and the 7900HT Fast RT-PCR System (Applied Biosystems) using SYBR Green PCR Master Mix (Life Technologies) and gene-specific primers. Relative expression levels were normalized to glyceraldehyde-3-phosphate dehydrogenase (GAPDH). Primers used for qPCR analysis are the following:

Cdh1_fwCAGTGAAGCGGCATCTAAAGC

Cdh1_revTTGGATTCAGAGGCAGGGTCG

Zeb1_fwCACAGTGGAGAGAAGCCATAC

Zeb1_revCACTGAGATGTCTTGAGTCCTG

Slug_fwCTCACCTCGGGAGCATACAG

Slug_revGACTTACACGCCCCAAGGATG

Snai1_fwCACACTGGTGAGAAGCCATTC

Snai1_revCTTGTGGAGCAAGGACATGCG

Krt14_fwCAGCAAGACAGAGGAGCTGAACC

Krt14_revCCAGGGATGCTTTCATGCTGAG

Krt18_fwGGTCTCAGCAGATTGAGGAGAG

Krt18_revCCAAGTCAATCTCCAAGGTCTGG

FGFR1_fwGGAGGCTACAAGGTCCGTTA

FGFR1_revTGCAGGTGTAGTTGCCCTTG

CDH1_fwCAGTCAAAAGGCCTCTACGG

CDH1_revCAGAAACGGAGGCCTGATGG

VIM_fwGGAAATGGCTCGTCACCTTC

VIM_revGAAATCCTGCTCTCCTCGCC

GLI1_fwCACAAGTGCACGTTTGAAGGG

GLI1_revCATGTATGGCTTCTCACCCG

CDH11_fwCGCAGAGCGTATACCAGATG

CDH11_revCCTCCTGTGTTTCATAGTCCG

TRIM32_fwCAGTTAACGTGGAAGATTCC

TRIM32_revGAGGCACTGCTGGATATTGG

### Plasmids, lentivirus production, and CRISPR mutations

KIF3A and IFT20 guide RNAs were selected from http://crispr.mit.edu/ and cloned into lentiCRIPRV2 plasmids containing either a puromycin resistance gene or a blasticidin resistance gene designed to replace the puromycin one. GLIS2 full-length and C-terminal (c. 1_444del) were amplified from a Precision LentiORF GLIS2 plasmid (Horizon Discovery) and cloned in frame with 3xHA or dTomato tags in a pEF_BSD lentiviral plasmid through GIBSON cloning. K251R mutation was introduced into GLIS2 by GIBSON cloning using the pEF_HA-GLIS2_BSD plasmid. FGFR1 was amplified from pCMV-SPORT6-FGFR1 plasmid (Horizon Discovery) and cloned in the LV-GFP plasmid (Addgene) in which H2B was replaced by FGFR1. FGFR1dn construct was generated by GIBSON cloning using the LV-FGFR1-GFP plasmid.

The following primers were used:

CRISPR

sgCTL_fw GCTGATCTATCGCGGTCGTC

sgCTL_rev GACGACCGCGATAGATCAGC

sgKIF3A_fw GAAATCAATGTGCTACAAAC

sgKIF3A_rev GTTTGTAGCACATTGATTTC

sgIFT20_fw CCAGCAGACCATAGAGCTGA

sgIFT20_rev TCAGCTCTATGGTCTGCTGG

sgTRIM32#1_fw CACCGGCGGACACCATTGATGCTAC

sgTRIM32#1_rev AAACGTAGCATCAATGGTGTCCGCC

sgTRIM32#2_fw CACCGAACTCGTCTGCGGGAACTTA

sgTRIM32#2_rev AAACTAAGTTCCCGCAGACGAGTTC

sgTRIM32#3_fw CACCGGTCTGCCCCGGCAATTCTGC

sgTRIM32#3_rev AAACGCAGAATTGCCGGGGCAGACC

sgTrim32#1_fw CACCGGCGGACGCCATTGATGCTGC

sgTrim32#1_rev AAACGCAGCATCAATGGCGTCCGCC

sgmTrim32#3_fw CACCGGGCTGCCTCGGCAGTTCTGC

sgmTrim32#3_rev AAACGCAGAACTGCCGAGGCAGCCC

HA-GLIS2 cloning:

1_GTGTCGTGAgccaccatggtgTACCCATACGATGTTCCTGAC

2_GTCAGGAACATCGTATGGGTAcaccatggtggcTCACGACAC

3_GACGTTCCAGATTACGCTCACTCCCTGGACGAGCCG

4_CGGCTCGTCCAGGGAGTGAGCGTAATCTGGAACGTC

5_GACGTTCCAGATTACGCTTTCCTTACCCCTCCCAAGGAC

6_GTCCTTGGGAGGGGTAAGGAAAGCGTAATCTGGAACGTC

7_CTCAAACCGGCTGTGGTGAACGGATCCGGCGCAACAAACTTC

8_GAAGTTTGTTGCGCCGGATCCGTTCACCACAGCCGGTTTGAG

GLIS2Cter cloning:

1_CTCAAACCGGCTGTGGTGAACGGATCCGGCGCAACAAACTTC

2_GAAGTTTGTTGCGCCGGATCCGTTCACCACAGCCGGTTTGAG

3_ctctggttatgtgtgggagggctaagaattcgttccggagtcgtcg

4_cgacgactccggaacgaattcttagccctcccacacataaccagag

5_ggatctggagcaacaaacttcACCTTCCTTACCCCTCCCAAGGAC

6_GTCCTTGGGAGGGGTAAGGAAGGTgaagtttgttgctccagatcc

HA-GLIS2K251R cloning:

1_CGGCTCGTCCAGGGAGTGAGCGTAATCTGGAACGTCATATGG

2_CCATATGACGTTCCAGATTACGCTCACTCCCTGGACGAGCCG

3_CGGTTGTGGATCCTCAGGTTCTC

4_GAGAACCTGAGGATCCACAACCG

5_CTCAAACCGGCTGTGGTGAACGGATCCGGCGCAACAAACTTC

6_GAAGTTTGTTGCGCCGGATCCGTTCACCACAGCCGGTTTGAG

FGFR1/FGFR1dn cloning:

1_GGACTCAAACGCCGCGATCCACCGGTCGCCACCGTGAGCA

2_GAGGCACTTCCAGCTCCACATggtggcccccctggggaga

3_tctccccaggggggccaccATGTGGAGCTGGAAGTGCCTC

4_TGCTCACGGTGGCGACCGGTGGATCGCGGCGTTTGAGTCC

5_tctccccaggggggccaccATGGTGAGCAAGGGCGAGG

6_CCTCGCCCTTGCTCACCATggtggcccccctggggaga

7_AGCTGCCTCGGGACAGAGATCCACCGGTCGCCACC 3’

8_GGTGGCGACCGGTGGATCTCTGTCCCGAGGCAGCT 5’

9_tctccccaggggggccaccATGGTGAGCAAGGGCGAGG

10_CCTCGCCCTTGCTCACCATggtggcccccctggggaga

11_GGATCACTCTCGGCATGGACGAGCTGTACAAGTAAatgc

12_gcatTTACTTGTACAGCTCGTCCATGCCGAGAGTGATCC

Lentiviruses were produced in 293FT cells after their transfection with lentiCRISPR or pEF_BSD_HA-GLIS2 or pEF_BSD_dTomGLIS2Cter (transfer) in combination with psPAX2 (packaging) and pMD2.G (envelope) plasmids, using Lipofectamine 2000 (Life Technologies) according to the manufacturer’s instructions. Supernatants containing lentiviruses were collected 48 and 72 hours after transfection. Primary mammary epithelial cells, HMLE, and HMLER cells growing in a monolayer were transduced with the supernatants containing viruses in the presence of polybrene (8 μg/ml). Transduced cells were selected with puromycin (2 μg/ml; GIBCO) or blasticidin (8 μg/ml; GIBCO). HMLE *KIF3A^−/−^* and *IFT20^−/−^* clones were selected by FACS.

### Gene expression and ChIP-seq analyses

For gene expression analysis in HMLE and HMLER cells, total RNA was extracted from highly confluent cells. Twenty-five nanograms for each of the two biological replicates per condition was used to generate libraries for whole-transcriptome analysis following High-Throughput 3′ Digital Gene Expression library preparation. Libraries were sequenced on Illumina Nextseq500. Raw sequence reads were collapsed by sequence identity and unique molecular identifier and aligned to the human genome (UCSC hg19 build) using TopHat v. 2.0.4. Genic read counts were quantified using the End Sequence Analysis Toolkit (v1). Count normalization and differential analyses were conducted using the DESeq package (R/Bioconductor), and genes with *q* < 0.05 and absolute fold change greater than 2 were tagged as differentially expressed. Row-normalized heatmaps for differentially expressed genes were created using Heatplus (R/Bioconductor). GSEAs were performed with the GSEA platform of the Broad Institute (www.broadinstitute.org/gsea/index.jsp). For gene expression analysis in Glis2 wild-type and mutant primary murine mammary epithelial cells, total RNA was extracted from sorted cells. The quality and quantity of RNA samples were determined using an Agilent Femto Pulse. RNA-seq libraries were prepared from 1 ng of total RNA using a TaKaRa SMARTer Stranded Total RNASeq Kit v2 Pico Input Mammalian kit with a 4-min fragmentation and 15 cycles of PCR. After cDNA cleanup, samples were treated with ZapR reagent and library fragments not cleaved by ZapR were further amplified by 15 cycles of PCR. Illumina libraries were validated using the Agilent Fragment Analyzer and quantified by qPCR, and 40-nucleotide (nt) paired-end reads were generated on Illumina NextSeq500. Paired-end RNA-seq reads were used to quantify gene expression Salmon (version 1.2.1) using a transcriptome derived from the mm10 primary assembly and an Ensembl v.100 annotation as a target. The mm10 genomic assembly was used as a selective alignment decoy. The resulting counts and transcript per million (TPM) values were assembled using tximport (version 1.18.0). The TPM values were transformed to log_2_ space with a plus 1 offset. Differential expression analysis was done using DESeq2 (version 1.30.0) and apeglm log fold change shrinkage. The heatmap was prepared using TIBCO Spotfire Analyst (version 7.11.2) and protein coding genes with an absolute log_2_ fold change ≥ 0.58 and adjusted *P* ≤ 0.05, rows were ordered by fold change, and columns were clustered with Ward’s method. Preranked GSEA (version 4.1.0) was run using the DESeq2 Wald statistic as a ranking metric and gene set collections from MSigDB (version 7.2). For gene expression analysis in breast tumors, total RNA was extracted from frozen sections of breast tumor samples [Rennes Biobank, bioresource research impact factor (BRIF) number: BB-0033-00056], using the NucleoSpin RNA set for a NucleoZOL kit according to the manufacturer’s protocol (Macherey-Nagel). Libraries were prepared using a modified version of a TaKaRa SMARTer Stranded Total RNA-Seq Kit Pico Input Mammalian kit. In brief, 100 ng of RNA at 10 ng/μl was sonicated using an RL230 Covaris sonicator (Covaris Inc.), and the resulting material was confirmed using the Fragment Analyzer (Agilent). Ten nanograms of each sonicated sample was prepared using a Pico Input kit as for formalin-fixed paraffin embedded (FFPE) samples using a 1:8 volume reduction on the SPT mosquito HV. Final libraries were validated by Fragment Analyzer and qPCR before sequencing on HiSeq2000 with 50-nt single-end reads. Reads were mapped to the human genome (USCS GRCh38/hg38 build), and transcript abundance was determined using STAR v. 2.7.2b. Molecular subtyping, PAM50 and claudin-low, was performed using the Genefu v.2.20.0 R package (www.pmgenomics.ca/bhklab/software/genefu). For gene expression analysis in mouse luminal cells and MaSC-basal cells, raw sequencing data for GSE60450 were downloaded from the Gene Expression Omnibus (GEO)/sequence read archive (SRA) repository (ftp-trace.ncbi.nlm.nih.gov/sra), and reads were mapped to the UCSC mm9 mouse genome build (genome.ucsc.edu) using RNA-seq by expectation-maximization (RSEM). Targeted pairwise differential expression analyses between basal and luminal samples were conducted using EBSeq v1.4.0 with median normalization. All RNA-seq analyses were conducted in the R Statistical Programming language. For gene expression analysis across breast tumor subtypes, the pipeline from Fougner *et al*. (*38*) was used on the TCGA-BRCA Dataset (github.com/clfougner/ClaudinLow). For ChIP-seq analysis, previously reported genome-wide murine Snail binding sites elucidated using ChIP-seq were obtained from the GEO (GSE61198). Mouse mm10 [National Center for Biotechnology Information (NCBI) Build 38/GRCm38 assembly] loci were translated to corresponding mm9 (NCBI Build 37 assembly) loci using the UCSC liftOver utility (command-line version timestamped 20170321). Peaks were annotated and labeled with corresponding genomic features using R package ChIPseeker (ver. 1.22.1) and UCSC mm9 knownGene annotation. Peaks within ±3000 base pairs of transcription sites were annotated as promoter-associated peaks. Mouse gene symbols were mapped to human equivalents using homology information from the Mouse Genome Informatics portal (informatics.jax.org), and promoter peaks from ChIP-seq analysis were subsequently associated with the 47 candidate ciliogenesis inducers along with differential gene expression results from Snail versus CTL RNA-seq analysis.

### Statistical analysis

Prism was used to analyze data, draw graphs, and perform statistical analyses. Data are presented as means ± SEM. Statistical analyses were carried out by Student’s *t* test unless otherwise specified. *P ≤ 0.05, **P ≤ 0.01, and ***P ≤ 0.001 were considered significant.

## Supplementary Materials

### This PDF file includes:

Figs. S1 to S8 Table S1 References

## Notes

View/request a protocol for this paper from *Bio-protocol*.
