# Supplementary material for: An EMT–primary cilium–GLIS2 signaling axis regulates mammogenesis and claudin-low breast tumorigenesis
Source: Sci Adv. 2021 Oct 27;7(44):eabf6063. doi: 10.1126/sciadv.abf6063 (PMC8550236; doi:10.1126/sciadv.abf6063)
Supplement: Supplementary file 1 — Figs. S1 to S8 Table S1 References [file sciadv.abf6063_sm.pdf]

Science Advances
AAAS

Supplementary Materials for

An EMT–primary cilium–GLIS2 signaling axis regulates mammogenesis and claudin-low breast tumorigenesis

Molly M. Wilson, Céline Callens, Matthieu Le Gallo, Svetlana Mironov, Qiong Ding, Amandine Salamagnon, Tony E. Chavarria, Roselyne Viel, Abena D. Peasah, Arjun Bhutkar, Sophie Martin, Florence Godey, Patrick Tas, Hong Soon Kang, Philippe P. Juin, Anton M. Jetten, Jane E. Visvader, Robert A. Weinberg, Massimo Attanasio, Claude Prigent, Jacqueline A. Lees, Vincent J. Guen*

*Corresponding author. Email: vincent.guen@univ-nantes.fr

Published 27 October 2021, *Sci. Adv.* **7**, eabf6063 (2021)
DOI: 10.1126/sciadv.abf6063

This PDF file includes:

Figs. S1 to S8
Table S1
References

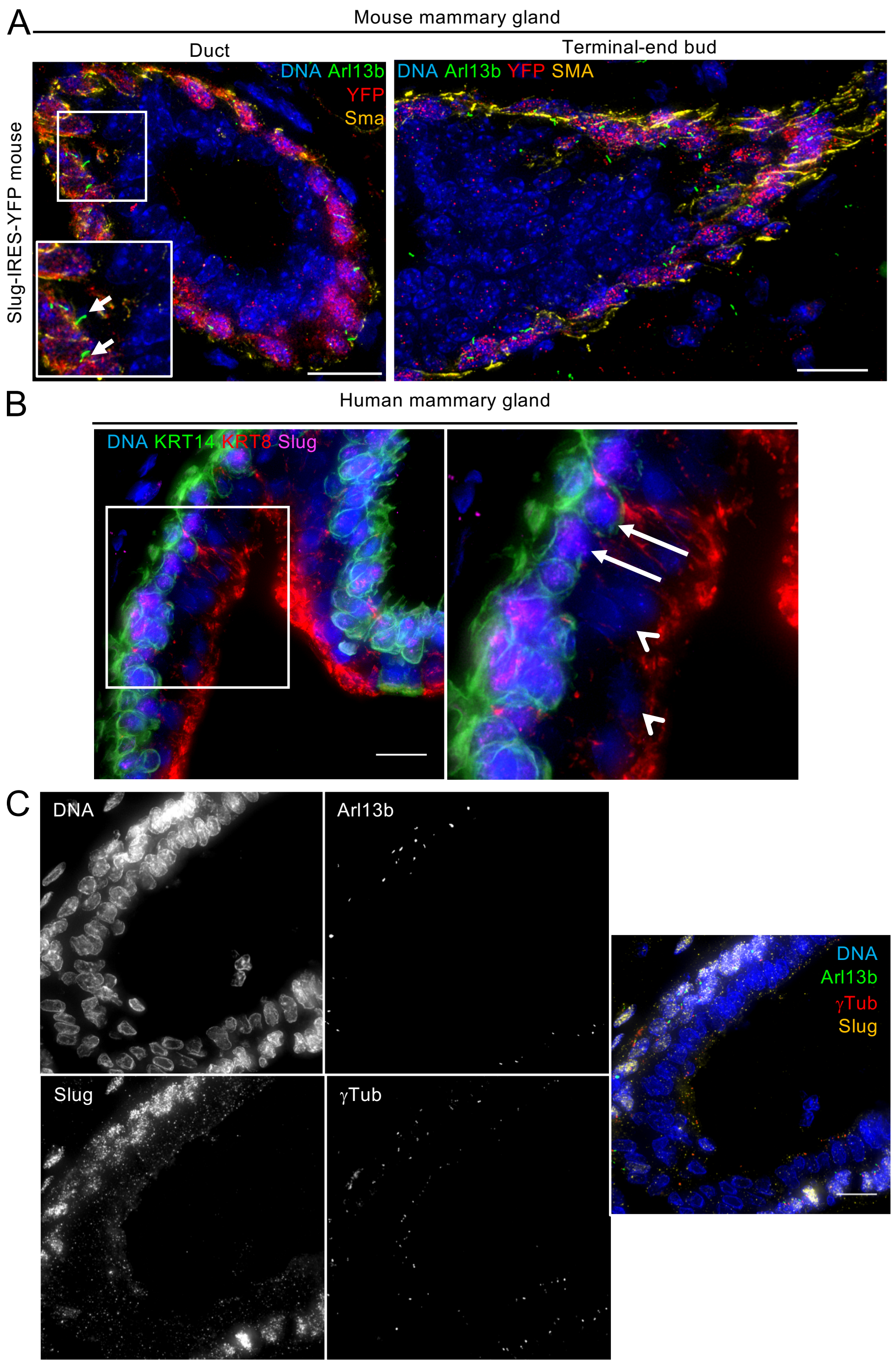

Fig. S1: EMT program activation and ciliogenesis in the mammary gland. (A) Mammary gland sections from Slug-IRES-YFP mice (8 weeks old) were stained for the indicated proteins. Representative images of a duct and of a remaining terminal-end bud (TEB) are shown. Scale bar: 15 μm (inset: 1.5X). (B, C) Mammary gland sections from healthy human patients were stained for the indicated proteins. Arrows indicate $Slug^{+}Krt14^{+}$ cells and arrowheads indicate $Slug^{-}Krt8^{+}$ cells. Scale bar: 15 μm, (inset: 2X).

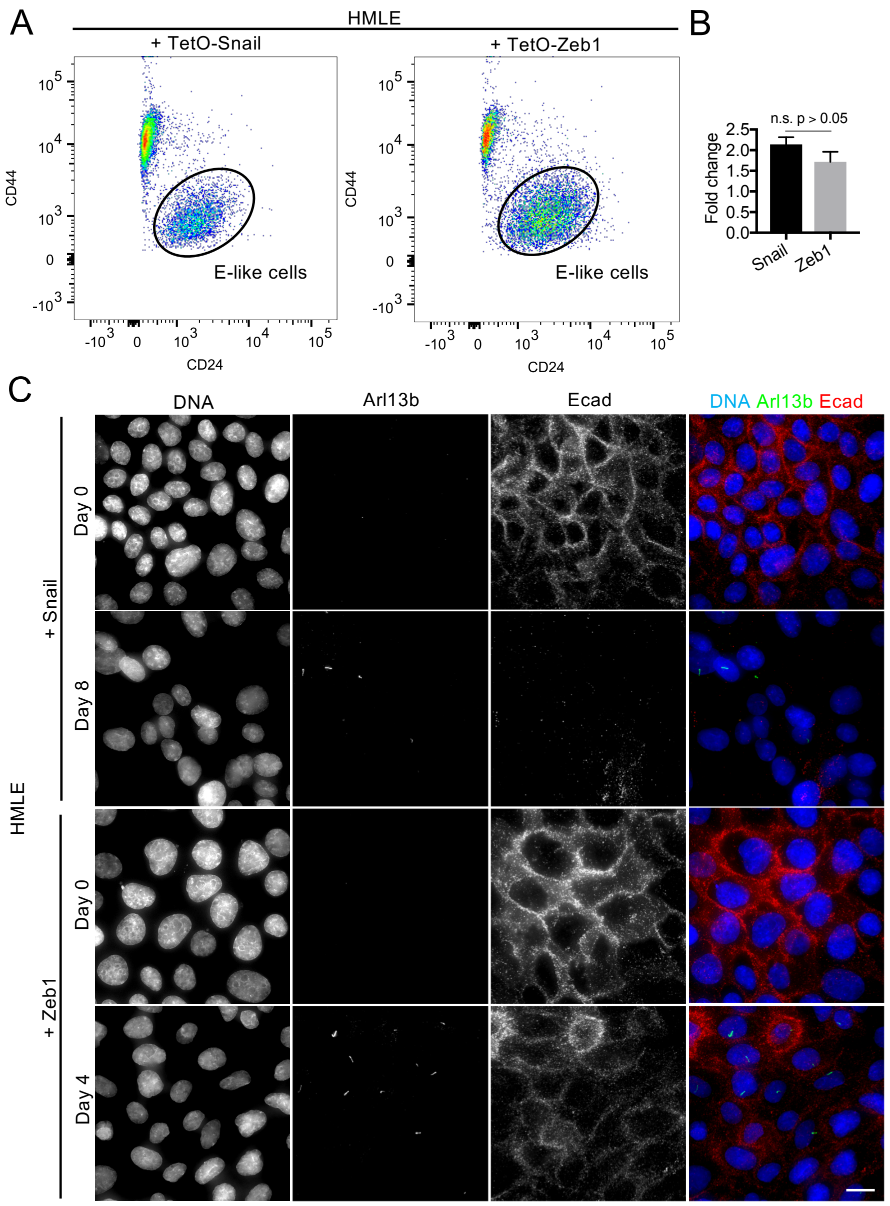

Fig. S2: EMT program activation and ciliogenesis in HMLE cells. (A) Epithelial-like (E-like, CD44^{lo} CD24^{hi}) cells from the indicated un-induced HMLE variants were isolated by FACS using the indicated cell surface markers. (B, C) Snail or Zeb1 expression was induced in Epithelial-like HMLE cells (as described in the legend of Figure 1). (B) Expression of both proteins was analyzed by western blot and fold change in protein expression between day 0 and 4 was quantified (n = 3, mean ± SEM). Representative results from 3 independent experiments are shown. (C) EMT and primary ciliogenesis was analyzed by immunofluorescence staining of the indicated proteins at the indicated time points. Scale bar: 15 μm. Representative results from 3 independent experiments are shown.

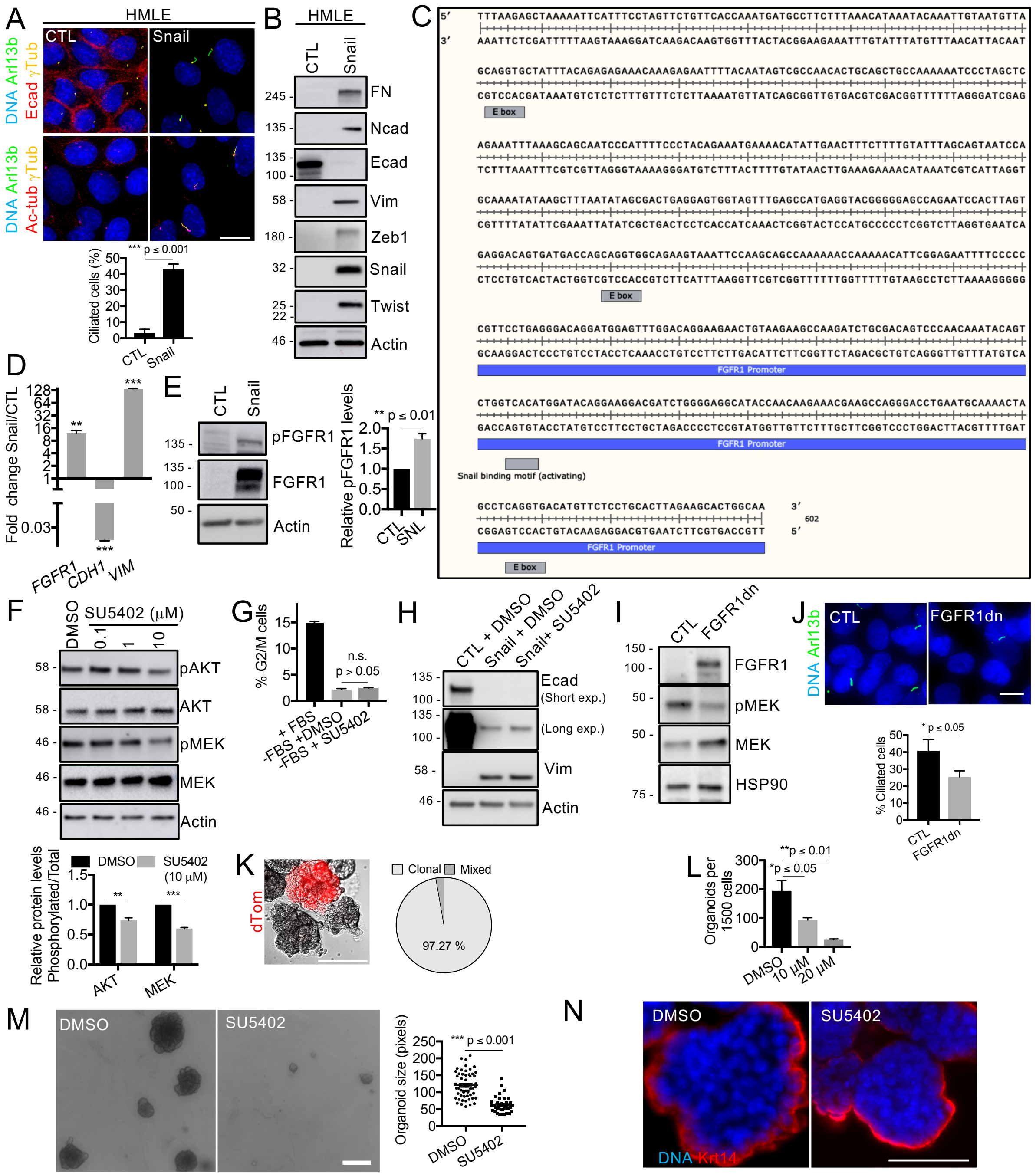

Fig. S3: EMT programs induce FGFR1 expression and FGF signaling controls MaSC stemness. (A) Control (CTL) and Snail-expressing HMLE cells were grown until high-confluence, serum starved for 24 h, and stained for the indicated proteins. The percentage of ciliated cells was measured (n = 3, mean ± SEM). Representative results from 3 independent experiments are shown (Scale bar: 15 μm). (B) Western blot analysis of the protein expression levels of EMT markers in CTL or Snail-expressing cells. (C) Snail-binding motifs associated to Snail dependent regulation of transcription (E-boxes or a binding motif associated to Snail-dependent activation of transcription(*44*)) were found in close proximity (within the 400 bp upstream) or in the *Fgfr1* promoter (mm9 genome). (D) Relative levels of the indicated gene transcripts were determined by real-time qPCR analysis of CTL and Snail-expressing HMLE

cells (n = 3, mean ± SEM). Representative results of 3 independent experiments are shown. (E) Western blot analysis of pFGFR1 levels in CTL and Snail-expressing HMLE cells. Phosphorylated protein levels were quantified (n = 3, mean ± SEM). Representative results of 3 independent experiments are shown. (F) Western blot analysis of MEK and AKT phosphorylation in Snail-expressing HMLE cells treated with DMSO or SU5402 at the indicated concentrations. Phosphorylated and total protein levels were quantified (n = 3, mean ± SEM). Representative results of 3 independent experiments are shown. (G) Snail-expressing HMLE cells were exposed to serum or serum-starved with DMSO or SU5402 (10 μM). The percentage of cells in G2/M was determined by FACS analysis of the DNA content in each variant (n = 3, mean ± SEM). Representative results of 3 independent experiments. (H) Western blot analysis of the protein expression levels of EMT markers in CTL or Snail-expressing cells treated with DMSO or with SU5402 (10 μM). (I, J) HMLE + Snail cells were transduced for expression of EGFP (CTL) or FGFR1-dominant negative-EGFP (FGFR1dn). (I) Western blot analysis of FGFR1dn, MEK, and pMEK expression was performed and (J) primary ciliogenesis was analyzed by immunofluorescence staining of the indicated protein. The percentage of ciliated cells was quantified (n = 3, mean ± SEM). Representative results from 3 independent experiments are shown. Scale bar: 15 μm. (K) MaSC-enriched basal cells that expressed dTomato (dTom) were mixed (1:1) with cells that did not express dTom and plated in 3D. The clonality of the organoid resulting from the co-culture was quantified by brightfield and fluorescence microscopy. Scale bar, 100 μm. (L, M) Organoid-forming capacity was determined for MaSC-enriched basal cells treated with SU5402 at the indicated concentrations 10 days after plating. Organoid number (L) and size (M) was quantified (n = 3, mean ± SEM). Representative results of 3 independent experiments. ** $p \leq 0.01$, *** $p \leq 0.001$. (N) The impact of SU5402 treatment on basal cell fate determination was analyzed by staining DMSO- or SU5402-treated organoids for Krt14. Scale bar: 50 μm.

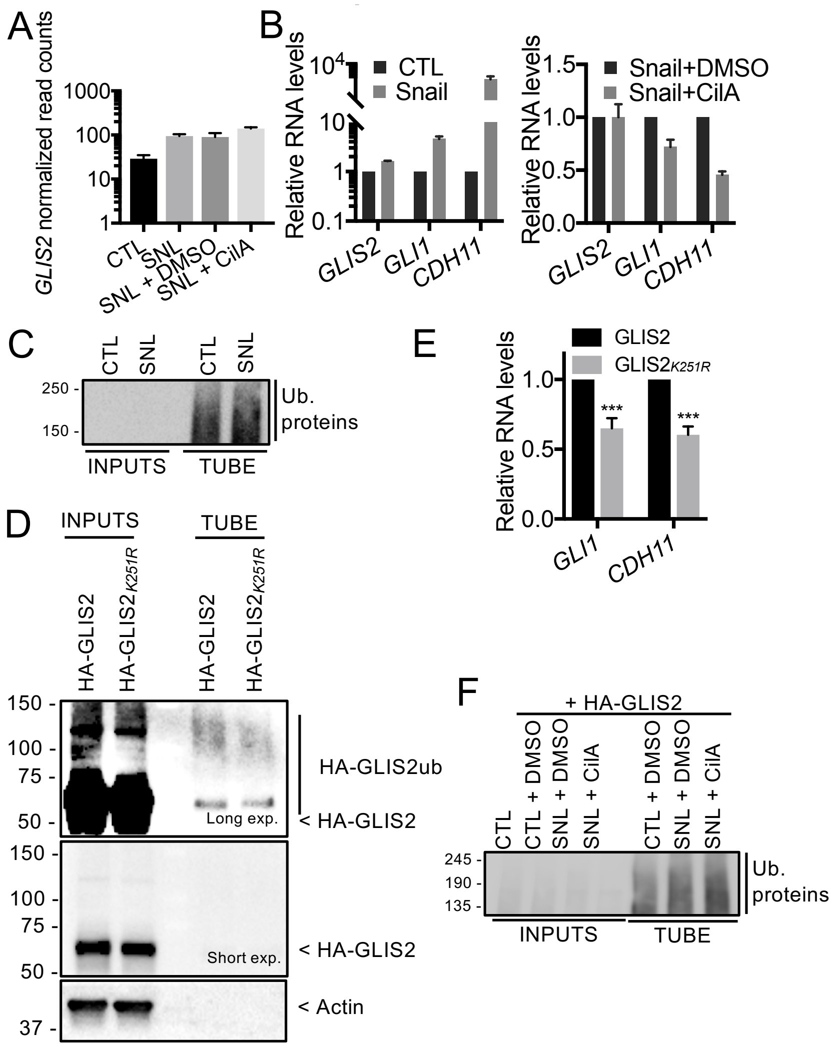

Fig. S4: GLIS2 inhibition occurs at the post-transcriptional level and is associated with ubiquitination. (A) Expression of *GLIS2* was identified in the indicated HMLE variants by RNA-sequencing as described in the legend of Fig. 3 (mean ± SEM). (B) Relative levels of the indicated gene transcripts in the indicated cells were determined by real-time qPCR analysis (n = 3, mean ± SEM). Representative results of 3 independent experiments. (C) CTL and Snail-expressing (SNL) HMLE cells were grown as described in the legend of Fig. 3. Ubiquitinated proteins were purified by TUBE pull-down and analyzed by western blot. (D, E) HA-GLIS2 or HA-GLIS2$_{K251R}$ were expressed in Snail-expressing HMLE cells, ubiquitinated proteins were purified by TUBE pull-down and analyzed by western blot (D), relative levels of the indicated gene transcripts in the indicated cells were determined by real-time qPCR analysis (n = 3, mean ± SEM), *** $p \leq 0.001$ (E). (F) CTL and Snail-expressing (SNL) HMLE cells with or without HA-GLIS2 were treated or not with DMSO and Ciliobrevin A (CilA). Ubiquitinated proteins were purified by TUBE pull-down and analyzed by western blot.

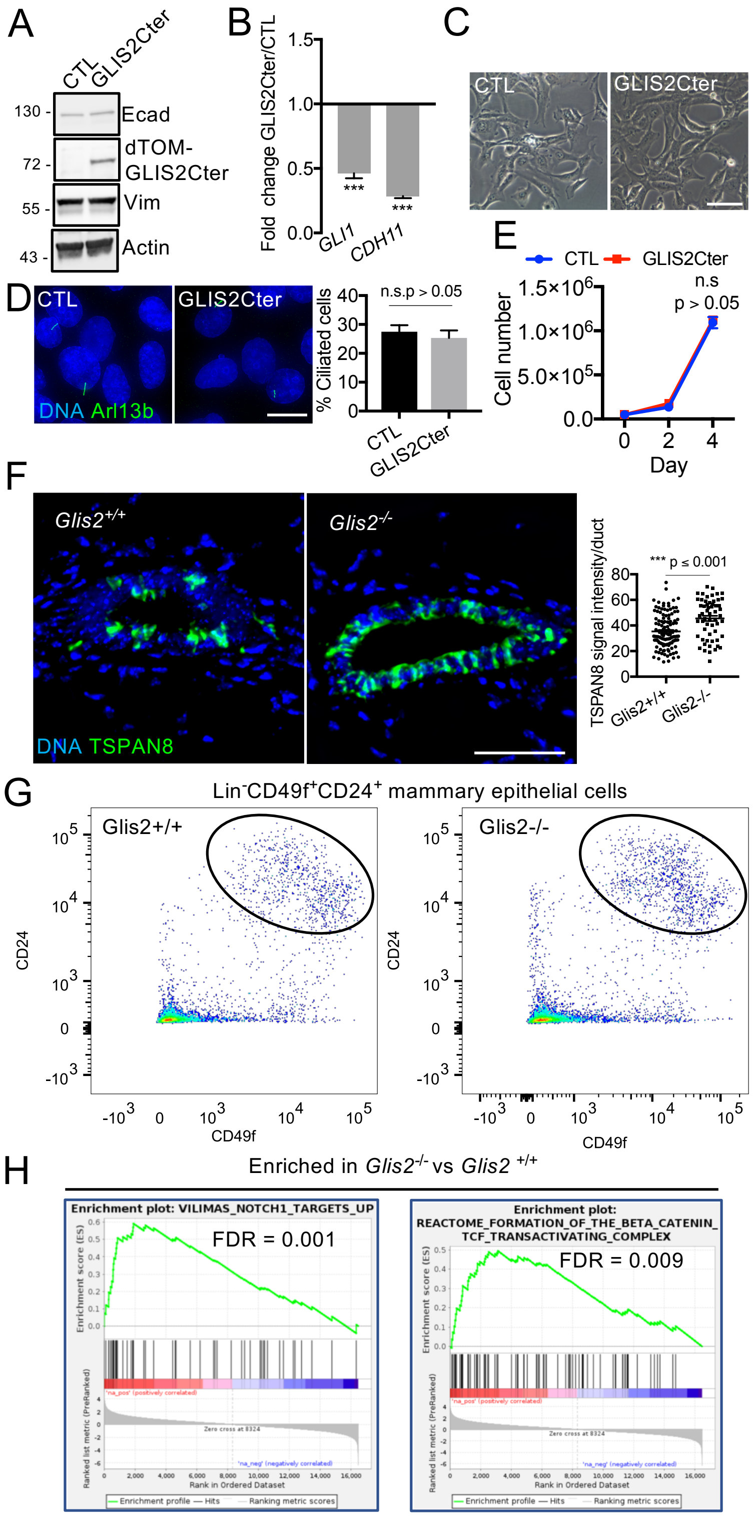

Fig. S5: Impact of GLIS2Cter expression or Glis2 deletion on EMT, primary ciliogenesis, cell signaling**.** (A) GLIS2Cter expression and its impact on expression of EMT markers in Snail-expressing HMLE cells were determined by western blot analysis. (B) Relative levels of *GLI1* and *CDH11* were determined in the indicated variants by real-time qPCR analysis (n = 3, mean ± SEM). Representative results of 3 independent experiments. (C-E) The impact of GLIS2Cter expression on morphology was examined by brightfield microscopy (C), on ciliogenesis was

assessed by immunofluorescence microscopy (D), on proliferation in 2-dimension was analyzed by counting the total cell number per well (3 wells/line) at the indicated time points (E). Scale bars: Brightfield 100 μm, Immunofluorescence 15 μm. (F) Paraffin sections from the mammary glands were stained with H&E or for the indicated protein. TSPAN8 signal intensity was measured (n = 3, mean ± SEM). Scale bar: 50 μm. (G) Mammary epithelial cells (Lin-CD49f+CD24+) were isolated by FACS from freshly dissociated mammary glands from 4 weeks old mice of the indicated phenotype. (H) Gene expression analysis and GSEA was performed as described in the legend of Fig. 4G, H.

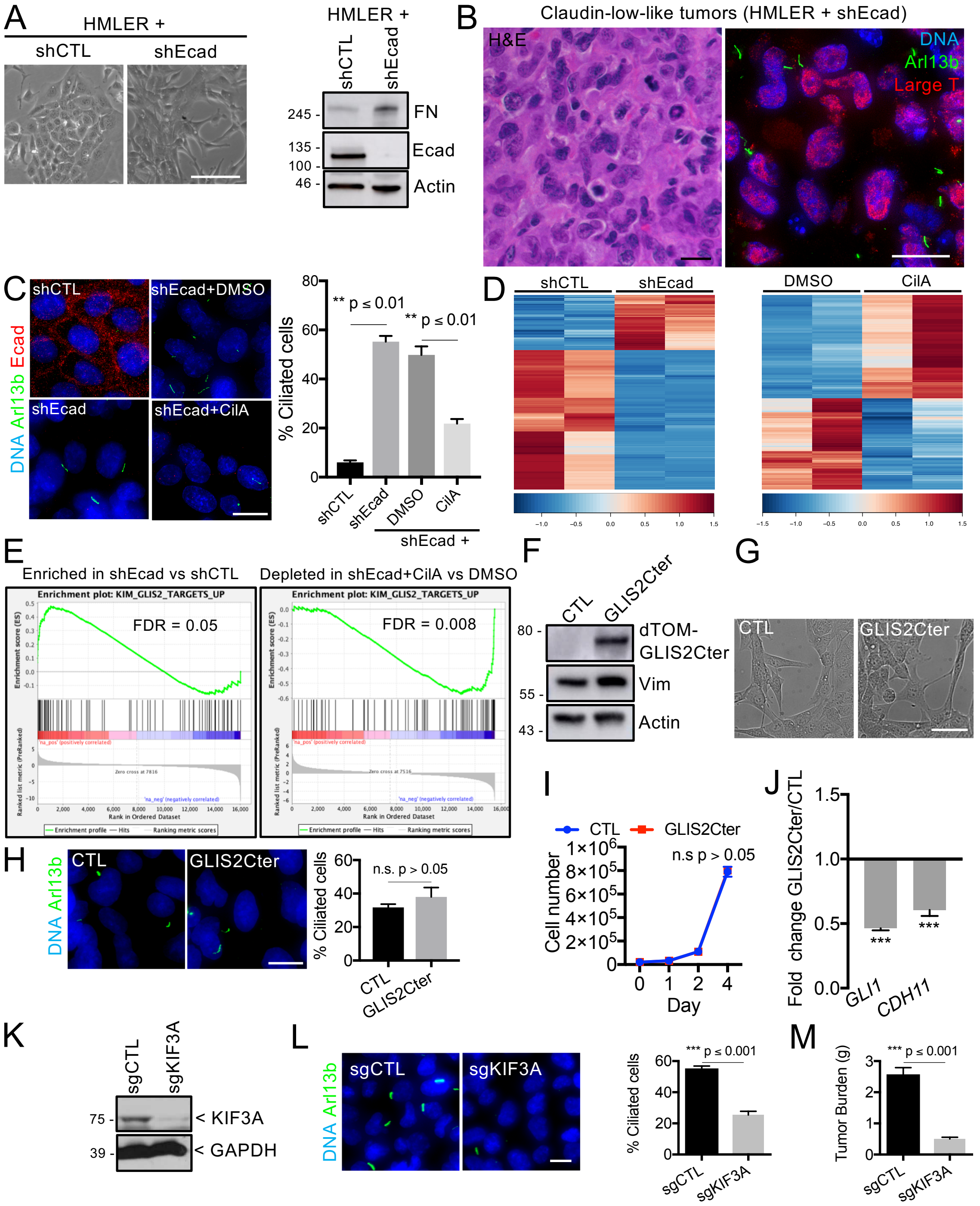

Fig. S6: EMT, primary ciliogenesis, and ciliary signaling in MaTIC biology. (A) shControl (CTL)- and sh-Ecadherin (Ecad)-expressing HMLER cells were examined for morphology by brightfield microscopy (scale bar: 100 µm) and for their expression of EMT markers by western blot analysis. (B) Sections of tumors arising from HMLER + shEcad cells that were orthotopically transplanted in the fat pad of female mice, as described in the legend of Fig. 5, were stained with H&E as well as for the indicated proteins. Representative images are shown. Scale bars: 15 µm. (C, D) shCTL- and shEcad-expressing HMLE cells were grown until high confluence and serum starved for 24h without or with DMSO or Ciliobrevin A (CilA). (C) Cells

were stained for the indicated proteins and the percentage of ciliated cells was determined (n = 3 mean ± SEM). Representative results from 3 independent experiments are shown. Scale bar: 15 μm. (D) Gene expression in the indicated HMLER variants was analyzed by RNA-sequencing. Heat-maps showing the differentially expressed genes (q-value ≤ 0.05, Fold-change ≥ 2) between samples are displayed. (E) A GSEA analysis shows significant enrichment of GLIS2 target genes in the list of upregulated genes in shEcad- versus (vs) shCTL-expressing cells (left panel) and depletion in the list of shEcad CilA-treated vs DMSO-treated cells (right panel). (F-J) The impact of GLIS2Cter expression in HMLER + shEcad cells on expression of EMT markers and GLIS2 targets, on morphology, on ciliogenesis and cell proliferation was examined as described in the legend of Fig. S5. Scale bars: Brightfield 50 μm, Immunofluorescence 15 μm. (K-M) The impact of *KIF3A* mutation in HMLER + shEcad cells on expression of KIF3A protein was analyzed by western blot (K), on ciliogenesis by immunofluorescence staining of the indicated protein (L, Scale bar: 15 μm, n = 3, mean ± SEM), on tumorigenesis (M) as described in the legend of Fig. 5D.

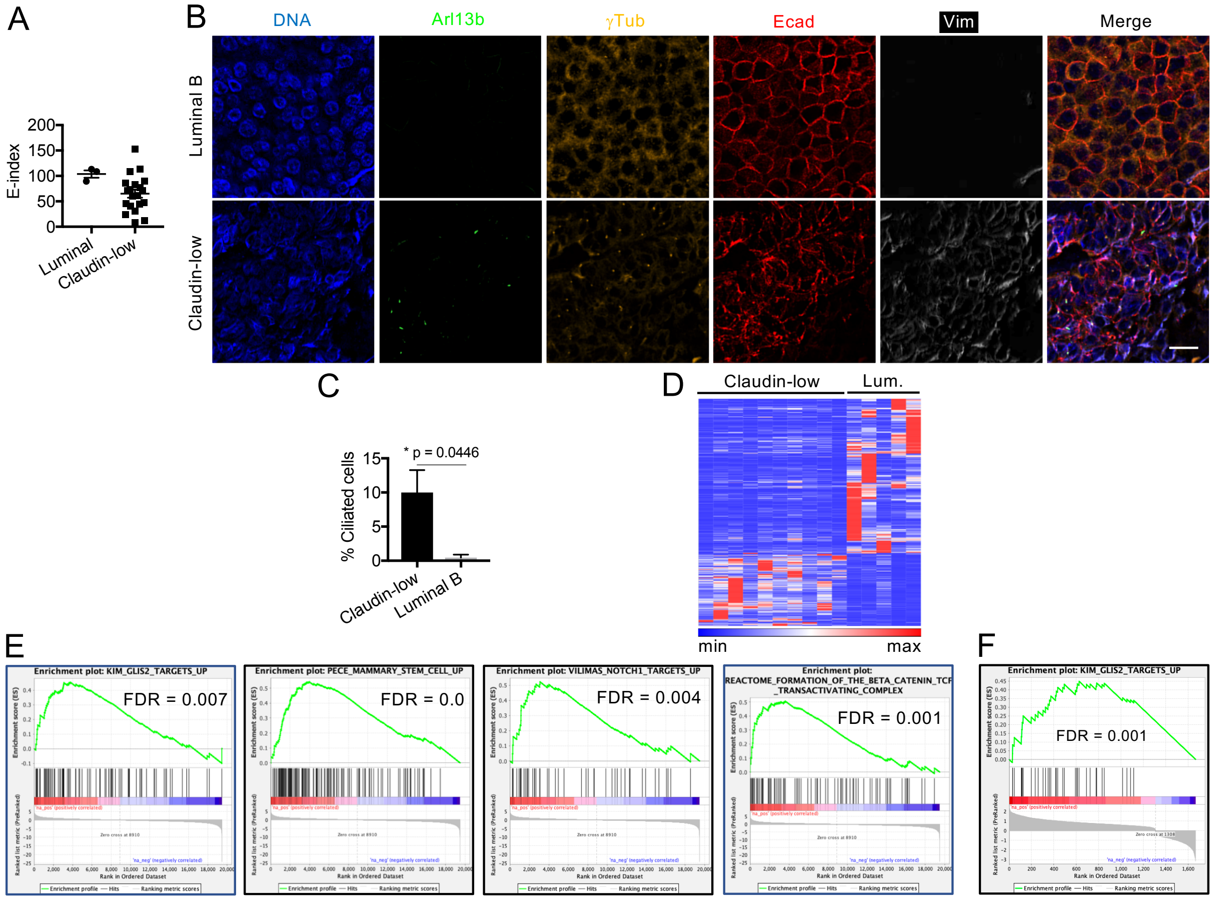

Fig. S7: Claudin-low tumors express low levels of epithelial markers, display primary cilia, express high levels of GLIS2 target genes, and of genes that mark MaSCs and activation of stem cell signaling pathways. (A-C) Paraffin sections of breast tumors were stained as described in the legend of Fig. 6B-C. (A) Ecadherin, Claudin 4 and Claudin 7 signal intensities were measured and used to define an Epithelial-index (E-index = (Ecadherin signal intensity + Claudin 4 signal intensity + Claudin 7 signal intensity)/3). (B) Scale bar: 20 μm. (C) The percentage of ciliated cells in Ecad+/Vim+ cancer cells in claudin-low tumors and in Ecad+ tumor cells in luminal tumors was quantified (n = 3, mean ± SEM). (D) Gene expression was analyzed in a subset of the claudin-low tumors identified in Fig. 6 and in luminal B tumors by RNA-sequencing. The heat-map showing the differentially expressed genes (q-value ≤ 0.05, Fold-change ≥ 2) between samples is displayed. (E) GSEA analysis shows significant enrichment of GLIS2 target genes, and of genes that mark MaSCs, EMT, Wnt pathway activation, Notch pathway activation, in the list of upregulated genes in claudin-low vs luminal tumors. (F) A GSEA analysis shows significant enrichment of GLIS2 target genes in the list of upregulated genes in claudin-low cancers versus other breast cancers defined by Prat et al (*37*).

A

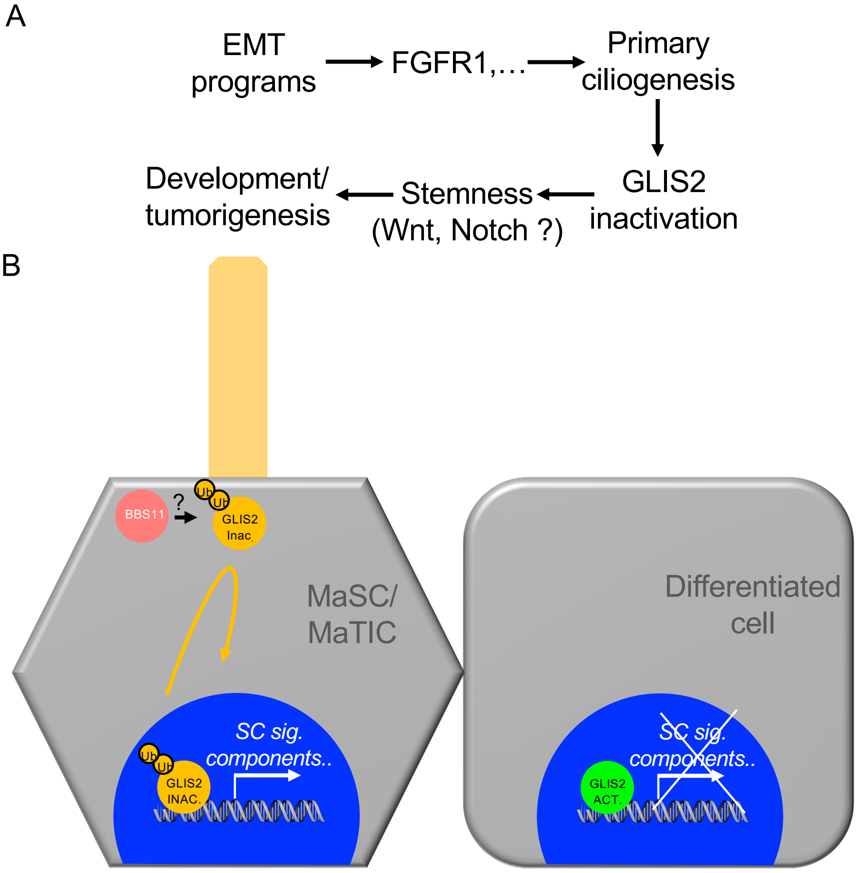

Fig. S8: Schematic representation of the EMT-primary cilium-GLIS2 axis in mammary epithelial cells. (A) Our results show that in MaSCs and MaTICs, activation of EMT programs directly induces expression of ciliogenesis inducers, including FGFR1, to promote ciliogenesis. The resulting primary cilia control inactivation of GLIS2 to regulate stemness of MaSC and development, as well as stemness of MaTIC and tumorigenesis. (B) Our results suggest that the primary cilium serves as a signaling platform where GLIS2 is ubiquitinated to be inactivated.

Category	Gene	Mean_CTL	Mean_Snail	FoldChange	log2FC	p-value	padj	Peak_Locus	Peak_score	TSS_dist
Centrosomal and/or cilium components with a function in ciliogenesis reported	FGFR1	13,97	350,12	25,07	4,65	3,30E-49	4,00E-47	chr8:26628989-26629969	967,08	0
	SYNE1	43,00	226,48	5,27	2,40	1,29E-10	2,06E-09	chr10:5151497-5152354	878,99	0
	TTLL1	18,34	93,76	5,11	2,35	1,65E-08	2,03E-07	chr15:83340984-83341547	389,4	0
	FUZ	5,07	24,88	4,90	2,29	0,004034	0,017176	chr7:52150597-52151812	75,41	0
	PARVA	148,44	540,71	3,64	1,86	2,12E-18	6,38E-17	chr7:119570608-119572212	1588,34	0
	ASAP1	127,56	460,95	3,61	1,85	1,55E-16	4,05E-15	chr15:64143186-64144290,chr15:64212826-64216500	127.2,85.76	0,0
	RAB8B	60,46	195,25	3,23	1,69	4,92E-06	4,16E-05	chr9:66766875-66767995	993,37	0
	DZIP1L	15,32	44,53	2,91	1,54	0,007533	0,029316	chr9:99532602-99533020	66,28	2340
	RILPL2	70,45	190,75	2,71	1,44	5,21E-07	5,17E-06	chr5:124927672-124928586	322,8	0
	ARL2BP	124,78	326,36	2,62	1,39	2,47E-09	3,41E-08	chr8:97189964-97191328	710,71	0
	INVS	30,32	78,14	2,58	1,37	0,001116	0,005545	chr4:48291980-48293202	961,25	0
	CEP19	34,32	87,71	2,56	1,35	0,000693	0,003625	chr16:32099007-32100345	1188,92	0
	BBS9	22,97	54,62	2,38	1,25	0,008213	0,031571	chr9:22279772-22280586	492,63	0
	NEK1	176,67	415,33	2,35	1,23	5,99E-05	0,000403	chr8:63471208-63472731	1219,21	0
	RAB23	83,61	186,13	2,23	1,15	5,21E-05	0,000355	chr1:33776383-33777461	897,16	0
	ARL3	603,47	1305,27	2,16	1,11	8,46E-11	1,38E-09	chr19:46647205-46648515	1301,37	0
	IFT52	158,15	339,10	2,14	1,10	8,92E-07	8,56E-06	chr2:162842567-162843527	798,53	0
	IFT122	34,14	71,23	2,09	1,06	0,010929	0,040142	chr6:115851028-115851446	62,39	-1202
	TULP3	145,98	301,64	2,07	1,05	9,58E-06	7,67E-05	chr6:128305242-128306816	899,44	0
	ICK	93,80	190,95	2,04	1,03	0,002504	0,011274	chr9:77956004-77957130,chr9:77960803-77961578	[illegible]83.94,368.9[illegible]	0,0
	GSN	751,96	1522,86	2,03	1,02	7,70E-10	1,12E-08	chr2:35111441-35112246	559,03	0
	HIF1A	436,14	857,24	1,97	0,97	7,93E-08	8,91E-07	chr12:75008273-75009778	1483,46	0
Centrosomal and/or cilium components with no function in ciliogenesis reported	HHIP	0,37	66,10	179,91	7,49	1,14E-06	1,08E-05	chr8:82581190-82582094	288,3	0
	MAP1B	31,35	1362,00	43,45	5,44	4,99E-80	1,46E-77	chr13:100217303-100217763,chr13:100284691-100287186	[illegible]8.57,1094.6[illegible]	-2881,0
	SPATA6	2,20	47,53	21,56	4,43	4,89E-09	6,50E-08	chr4:111392399-111393074	344,24	0
	ROM1	9,92	68,81	6,93	2,79	5,41E-08	6,22E-07	chr19:9002887-9005351	811,54	0
	CEP112	20,36	76,58	3,76	1,91	1,90E-05	0,000143	chr11:108286427-108286794	419,03	0
	CIB2	12,49	37,28	2,98	1,58	0,009003	0,034073	chr9:54407616-54408430	628,13	0
	PKD2	93,53	264,48	2,83	1,50	6,06E-09	7,95E-08	chr5:104887764-104889627	849,1	0
	PRKACB	66,59	168,72	2,53	1,34	6,92E-06	5,69E-05	chr3:146475485-146476580	884,49	0
	GLI3	68,69	165,75	2,41	1,27	2,03E-05	0,000152	chr13:15554405-15556571	631,33	0
	GABARAP	1633,26	3544,08	2,17	1,12	1,79E-13	3,74E-12	chr11:69804207-69805537	985,15	0
	AK1	392,72	785,77	2,00	1,00	7,78E-05	0,00051	chr2:32484191-32485622	220,98	0
	PRKAR1A	636,46	1249,94	1,96	0,97	7,29E-09	9,45E-08	chr11:109511449-109513326	615,97	0

Table S1: Genes that code for centrosomal/ciliary proteins are expressed at higher levels upon Snail expression and are Snail direct targets. Genes that code for centrosomal or ciliary components with (blue) or without (white) a reported function in ciliogenesis were identified as expressed at higher levels in Snail-expressing vs CTL HMLE cells. Snail binding sites were identified in the promoter region of the genes. Promoter peaks (+/- 3kbp) were identified by analyzing GSE61198 (Mean = Mean gene expression across samples; log2FC = log2 Fold Change; padj = adjusted p-value; TSS_dist = Distance of peak from transcription start site of gene).

REFERENCES AND NOTES

1. N. Y. Fu, E. Nolan, G. J. Lindeman, J. E. Visvader, Stem cells and the differentiation hierarchy in mammary gland development. *Physiol. Rev.* **100**, 489–523 (2020).
2. S. A. Mani, W. Guo, M. J. Liao, E. N. Eaton, A. Ayyanan, A. Y. Zhou, M. Brooks, F. Reinhard, C. C. Zhang, M. Shipitsin, L. L. Campbell, K. Polyak, C. Brisken, J. Yang, R. A. Weinberg, The epithelial-mesenchymal transition generates cells with properties of stem cells. *Cell* **133**, 704–715 (2008).
3. W. Guo, Z. Keckesova, J. L. Donaher, T. Shibue, V. Tischler, F. Reinhardt, S. Itzkovitz, A. Noske, U. Zürrer-Härdi, G. Bell, W. L. Tam, S. A. Mani, A. van Oudenaarden, R. A. Weinberg, Slug and Sox9 cooperatively determine the mammary stem cell state. *Cell* **148**, 1015–1028 (2012).
4. X. Ye, W. L. Tam, T. Shibue, Y. Kaygusuz, F. Reinhardt, E. Ng Eaton, R. A. Weinberg, Distinct EMT programs control normal mammary stem cells and tumour-initiating cells. *Nature* **525**, 256–260 (2015).
5. M. M. Wilson, R. A. Weinberg, J. A. Lees, V. J. Guen, Emerging mechanisms by which EMT programs control stemness. *Trends Cancer* **6**, 775–780 (2020).
6. M. Nassour, Y. Idoux-Gillet, A. Selmi, C. Côme, M. L. M. Faraldo, M. A. Deugnier, P. Savagner, Slug controls stem/progenitor cell growth dynamics during mammary gland morphogenesis. *PLOS ONE* **7**, e53498 (2012).
7. S. Phillips, A. Prat, M. Sedic, T. Proia, A. Wronski, S. Mazumdar, A. Skibinski, S. H. Shirley, C. M. Perou, G. Gill, P. B. Gupta, C. Kuperwasser, Cell-state transitions regulated by SLUG are critical for tissue regeneration and tumor initiation. *Stem Cell Rep.* **2**, 633–647 (2014).
8. N. Harbeck, F. Penault-Llorca, J. Cortes, M. Gnant, N. Houssami, P. Poortmans, K. Ruddy, J. Tsang, F. Cardoso, Breast cancer. *Nat. Rev. Dis. Primers* **5**, 66 (2019).
9. E. L. McCoy, R. Iwanaga, P. Jedlicka, N.S. Abbey, L. A. Chodosh, K. A. Heichman, A. L. Welm, H. L. Ford, Six1 expands the mouse mammary epithelial stem/progenitor cell pool and induces mammary tumors that undergo epithelial-mesenchymal transition. *J. Clin. Invest.* **119**, 2663–2677 (2009).
10. A. P. Morel, G. W. Hinkal, C. Thomas, F. Fauvet, S. Courtois-Cox, A. Wierinckx, M. Devouassoux-Shisheboran, I. Treilleux, A. Tissier, B. Gras, J. Pourchet, I. Puisieux, G. J. Browne, D. B. Spicer, J. Lachuer, S. Ansieau, A. Puisieux, EMT inducers catalyze malignant transformation of mammary

epithelial cells and drive tumorigenesis towards claudin-low tumors in transgenic mice. *PLOS Genet.* **8**, e1002723 (2012).

11. V. J. Guen, T. E. Chavarria, C. Kröger, X. Ye, R. A. Weinberg, J. A. Lees, EMT programs promote basal mammary stem cell and tumor-initiating cell stemness by inducing primary ciliogenesis and Hedgehog signaling. *Proc. Natl. Acad. Sci. U.S.A.* 114, E10532–E10539 (2017).
12. V. J. Guen, C. Prigent, Targeting primary ciliogenesis with small-molecule inhibitors. *Cell Chem. Biol.* **27**, 1224–1228 (2020).
13. Z. Anvarian, K. Mykytyn, S. Mukhopadhyay, L. B. Pedersen, S. T. Christensen, Cellular signalling by primary cilia in development, organ function and disease. *Nat. Rev. Nephrol.* **15**, 199–219 (2019).
14. Y. G. Han, N. Spassky, M. Romaguera-Ros, J.M. Garcia-Verdugo, A. Aguilar, S. Schneider-Maunoury, A. Alvarez-Buylla, Hedgehog signaling and primary cilia are required for the formation of adult neural stem cells. *Nat. Neurosci.* **11**, 277–284 (2008).
15. C. K. Tong, Y.G. Han, J. K. Shah, K. Obernier, C. D. Guinto, A. Alvarez-Buylla, Primary cilia are required in a unique subpopulation of neural progenitors. *Proc. Natl. Acad. Sci. U.S.A.* **111**, 12438–12443 (2014).
16. K. I. Hilgendorf, C. T. Johnson, A. Mezger, S. L. Rice, A. M. Norris, J. Demeter, W. J. Greenleaf, J. F. Reiter, D. Kopinke, P. K. Jackson, Omega-3 fatty acids activate ciliary FFAR4 to control adipogenesis. *Cell* **179**, 1289–1305.e21 (2019).
17. A. M. Joiner, W. W. Green, J. C. McIntyre, B. L. Allen, J. E. Schwob, J. R. Martens, Primary cilia on horizontal basal cells regulate regeneration of the olfactory epithelium. *J. Neurosci.* **35**, 13761–13772 (2015).
18. B. Elenbaas, L. Spirio, F. Koerner, M. D. Fleming, D. B. Zimonjic, J. L. Donaher, N. C. Popescu, W. C. Hahn, R. A. Weinberg, Human breast cancer cells generated by oncogenic transformation of primary mammary epithelial cells. *Genes Dev.* **15**, 50–65 (2001).
19. J. M. Neugebauer, J. D. Amack, A. G. Peterson, B. W. Bisgrove, H. J. Yost, FGF signalling during embryo development regulates cilia length in diverse epithelia. *Nature* **458**, 651–654 (2009).
20. A. Honda, T. Kita, S. V. Seshadri, K. Misaki, Z. Ahmed, J. E. Ladbury, G. P. Richardson, S. Yonemura, R. K. Ladher, FGFR1-mediated protocadherin-15 loading mediates cargo specificity during intraflagellar transport in inner ear hair-cell kinocilia. *Proc. Natl. Acad. Sci. U.S.A.* **115**, 8388–8393 (2018).

21. A. D. Jenks, S. Vyse, J. P. Wong, E. Kostaras, D. Keller, T. Burgoyne, A. Shoemark, A. Tsalikis, M. de la Roche, M. Michaelis, J. Cinatl Jr, P. H. Huang, B. E. Tanos, Primary cilia mediate diverse kinase inhibitor resistance mechanisms in cancer. *Cell Rep.* **23**, 3042–3055 (2018).
22. A. C. Pond, X. Bin, T. Batts, K. Roarty, S. Hilsenbeck, J. M. Rosen, Fibroblast growth factor receptor signaling is essential for normal mammary gland development and stem cell function. *Stem Cells* **31**, 178–189 (2013).
23. B. T. Spike, D. D. Engle, J. C. Lin, S. K. Cheung, J. la, G. M. Wahl, A mammary stem cell population identified and characterized in late embryogenesis reveals similarities to human breast cancer. *Cell Stem Cell* **10**, 183–197 (2012).
24. S. C. Goetz, K. V. Anderson, The primary cilium: A signalling centre during vertebrate development. *Nat. Rev. Genet.* **11**, 331–344 (2010).
25. A. J. Firestone, J. S. Weinger, M. Maldonado, K. Barlan, L. D. Langston, M. O'Donnell, V. I. Gelfand, T. M. Kapoor, J. K. Chen, Small-molecule inhibitors of the AAA+ ATPase motor cytoplasmic dynein. *Nature* **484**, 125–129 (2012).
26. M. Attanasio, N. H. Uhlenhaut, V. H. Sousa, J. F. O'Toole, E. Otto, K. Anlag, C. Klugmann, A.C. Treier, J. Helou, J. A. Sayer, D. Seelow, G. Nürnberg, C. Becker, A. E. Chudley, P. Nürnberg, F. Hildebrandt, M. Treier, Loss of GLIS2 causes nephronophthisis in humans and mice by increased apoptosis and fibrosis. *Nat. Genet.* **39**, 1018–1024 (2007).
27. Y. S. Kim, H. S. Kang, R. Herbert, J. Y. Beak, J. B. Collins, S. F. Grissom, A. M. Jetten, Kruppel-like zinc finger protein Glis2 is essential for the maintenance of normal renal functions. *Mol. Cell. Biol.* **28**, 2358–2367 (2008).
28. D. Singer, K. Thamm, H. Zhuang, J. Karbanová, Y. Gao, J.V. Walker, H. Jin, X. Wu, C.R. Coveney, P. Marangoni, D. Lu, P.R.C. Grayson, T. Gulsen, K.J. Liu, S. Ardu, A.K.T. Wann, S. Luo, A.C. Zambon, A.M. Jetten, C. Tredwin, O.D. Klein, M. Attanasio, P. Carmeliet, W.B. Huttner, D. Corbeil, B. Hu, Prominin-1 controls stem cell activation by orchestrating ciliary dynamics. *EMBO J.* **38**, e99845 (2019).
29. P. Holmfeldt, M. Ganuza, H. Marathe, B. He, T. Hall, G. Kang, J. Moen, J. Pardieck, A. C. Saulsberry, A. Cico, L. Gaut, D. McGoldrick, D. Finkelstein, K. Tan, S. McKinney-Freeman, Functional screen identifies regulators of murine hematopoietic stem cell repopulation. *J. Exp. Med.* **213**, 433–449 (2016).

30. H. Ramachandran, T. Schäfer, Y. Kim, K. Herfurth, S. Hoff, S. S. Lienkamp, A. Kramer-Zucker, G. Walz, Interaction with the Bardet-Biedl gene product TRIM32/BBS11 modifies the half-life and localization of Glis2/NPHP7. *J. Biol. Chem.* **289**, 8390–8401 (2014).
31. V. Akimov, I. Barrio-Hernandez, S. V. F. Hansen, P. Hallenborg, A.K. Pedersen, D. B. Bekker-Jensen, M. Puglia, S. D. K. Christensen, J. T. Vanselow, M. M. Nielsen, I. Kratchmarova, C. D. Kelstrup, J. V. Olsen, B. Blagoev, UbiSite approach for comprehensive mapping of lysine and N-terminal ubiquitination sites. *Nat. Struct. Mol. Biol.* **25**, 631–640 (2018).
32. Y. H. Kim, D. Epting, K. Slanchev, C. Engel, G. Walz, A. Kramer-Zucker, A complex of BBS1 and NPHP7 is required for cilia motility in zebrafish. *PLOS ONE* **8**, e72549 (2013).
33. A. V. Loktev, Q. Zhang, J. S. Beck, C. C. Searby, T. E. Scheetz, J. F. Bazan, D. C. Slusarski, V. C. Sheffield, P. K. Jackson, M. V. Nachury, A BBSome subunit links ciliogenesis, microtubule stability, and acetylation. *Dev. Cell* **15**, 854–865 (2008).
34. F. Zhang, *G*. Nakanishi, S. Kurebayashi, K. Yoshino, A. Perantoni, Y.S. Kim, A.M. Jetten, Characterization of Glis2, a novel gene encoding a Gli-related, Krüppel-like transcription factor with transactivation and repressor functions. Roles in kidney development and neurogenesis. *J. Biol. Chem.* **277**, 10139–10149 (2002).
35. N. Y. Fu, A. C. Rios, B. Pal, C. W. Law, P. Jamieson, R. Liu, F. Vaillant, F. Jackling, K. H. Liu, G. K. Smyth, G. J. Lindeman, M. E. Ritchie, J. E. Visvader, Identification of quiescent and spatially restricted mammary stem cells that are hormone responsive. *Nat. Cell Biol.* **19**, 164–176 (2017).
36. Y. S. Kim, H. S. Kang, A. M. Jetten, The Krüppel-like zinc finger protein Glis2 functions as a negative modulator of the Wnt/β-catenin signaling pathway. *FEBS Lett.* **581**, 858–864 (2007).
37. A. Prat, J. S. Parker, O. Karginova, C. Fan, C. Livasy, J. I. Herschkowitz, X. He, C. M. Perou, Phenotypic and molecular characterization of the claudin-low intrinsic subtype of breast cancer. *Breast Cancer Res.* **12**, R68 (2010).
38. C. Fougner, H. Bergholtz, J. H. Norum, T. Sorlie, Re-definition of claudin-low as a breast cancer phenotype. *Nat. Commun.* **11**, 1787 (2020).
39. M. Rozycki, M. Lodyga, J. Lam, M. Z. Miranda, K. Fátyol, P. Speight, A. Kapus, The fate of the primary cilium during myofibroblast transition. *Mol. Biol. Cell* **25**, 643–657 (2014).
40. X. Liu, Y. Wang, F. Liu, M. Zhang, H. Song, B. Zhou, C. W. Lo, S. Tong, Z. Hu, Z. Zhang, Wdpcp promotes epicardial EMT and epicardium-derived cell migration to facilitate coronary artery remodeling. *Sci. Signal.* **11**, eaah5770 (2018).

41. B. Li, A. A. Rauhauser, J. Dai, R. Sakthivel, P. Igarashi, A. M. Jetten, M. Attanasio, Increased hedgehog signaling in postnatal kidney results in aberrant activation of nephron developmental programs. *Hum. Mol. Genet.* **20**, 4155–4166 (2011).
42. V. J. Guen, C. Gamble, M. Flajolet, S. Unger, A. Thollet, Y. Ferandin, A. Superti-Furga, P. A. Cohen, L. Meijer, P. Colas, CDK10/cyclin M is a protein kinase that controls ETS2 degradation and is deficient in STAR syndrome. *Proc. Natl. Acad. Sci. U.S.A.* **110**, 19525–19530 (2013).
43. V. J. Guen, C. Gamble, D. E. Perez, S. Bourassa, H. Zappel, J. Gärtner, J. A. Lees, P. Colas, STAR syndrome-associated CDK10/cyclin M regulates actin network architecture and ciliogenesis. *Cell Cycle* **15**, 678–688 (2016).
44. W. S. Wu, R.I. You, C.C. Cheng, M.C. Lee, T.Y. Lin, C.T. Hu, Snail collaborates with EGR-1 and SP-1 to directly activate transcription of MMP 9 and ZEB1. *Sci. Rep.* **7**, 17753 (2017).
